# Diversity-Oriented Synthesis Based on the DPPP-Catalyzed Mixed Double-Michael Reactions of Electron-Deficient Acetylenes and β-Amino Alcohols

**DOI:** 10.3390/molecules16053802

**Published:** 2011-05-05

**Authors:** Yi Chiao Fan, Ohyun Kwon

**Affiliations:** Department of Chemistry and Biochemistry, University of California, Los Angeles, CA 90095, USA

**Keywords:** phosphine catalysis, Michael reaction, oxazolidines, β-amino alcohols, acetylenes

## Abstract

In this study, we prepared oxizolidines through 1,3-bis(diphenylphosphino)-propane (DPPP)–catalyzed mixed double-Michael reactions of β-amino alcohols with electron-deficient acetylenes. These reactions are very suitable for the diversity-oriented parallel syntheses of oxizolidines because: (i) they are performed under mild metal-free conditions and (ii) the products are isolated without complicated work-up. To demonstrate the applicability of mixed double-Michael reactions for the preparation of five-membered-ring heterocycles, we prepared 60 distinct oxazolidines from five β-amino alcohols and 12 electron-deficient acetylenes. We synthesized 36 of these 60 oxazolidines in enantiomerically pure form from proteinogenic amino acid–derived β-amino alcohols.

## 1. Introduction 

Because small organic heterocyclic compounds are found in most pharmaceuticals and agrochemicals and also in numerous natural products, additives, modifiers, and polymers [1,2], their construction remains of great interest to the synthetic organic chemistry community. In particular, the preparation of libraries of heterocyclic compounds through parallel combinatorial synthesis [3,4] or branched diversity-oriented synthesis [5,6,7] would be an attractive approach toward identifying biologically active small organic molecules [8,9,10]. Simple, robust, and metal-free methods for synthesizing small molecules are especially attractive if these compounds are to be used for biological testing. Furthermore, reactions involving organocatalysis are becoming increasingly desirable in terms of their low environmental impact. From these considerations, phosphine-catalyzed annulation has served as an ideal format for synthesizing diverse groups of heterocyclic small molecules with high efficiency and minimal impact on the environment [11,12,13,14,15,16,17,18,19,20,21,22,23,24,25,26,27,28,29]. In particular, 1,3-bis(diphenylphosphino)-propane (DPPP)–catalyzed double Michael addition has proved to be an atom-economical platform for preparing ten unique heterocyclic compounds [30,31].

A little over three decades ago, White and Baizer reported the first phosphine-catalyzed Michael additions of benzyl alcohol to activated olefins [32]. Only in the last two decades, however, did other synthetic research groups start studying Michael additions using nucleophilic phosphine catalysts and activated acetylenes. Inanaga demonstrated the first Michael additions of benzyl alcohol to activated acetylenes in the presence of nucleophilic phosphine catalysts [33]. When acetylenes are used as Michael acceptors, the Michael products possess one remaining degree of unsaturation, thereby enabling further incorporation of nucleophiles.

Capitalizing on the possibility of performing two consecutive Michael additions to activated acetylenes, Grossman elegantly demonstrated the double-Michael additions of carbon pro-nucleophiles to generate functionalized cyclohexanes under the influence of phosphine catalysts and bases [34,35]. Subsequently, Yavari employed catechol—an oxygen di-nucleophile—as the Michael donor [36]; although the yield of the resulting 1,3-benzodioxole was low (20%, isolated), he confirmed that heteroatom double-Michael addition under phosphine catalysis was possible [37]. Nevertheless, to this day, few heterocyclic compounds have been generated using double-Michael additions.

**Scheme 1:**
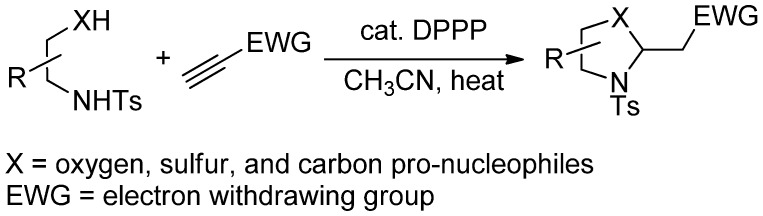
DPPP-Catalyzed Mixed Double-Michael Reactions.

To overcome poor efficiency and the inability to incorporate other heteroatoms such as nitrogen and sulfur, in the phosphine-catalyzed double-Michael reactions, we set out to develop a route to generate various heterocyclic compounds under general and robust phosphine catalysis conditions. By employing DPPP as the catalyst, we have synthesized functionalized oxazolidines, thiazolidines, and pyrrolidines in high efficiency (Scheme 1) [30]. By employing dinucleophiles tethered through aromatic rings, we have obtained indolines, dihydropyrrolopyridines, benzimidazolines, tetrahydroquinolines, tetrahydroisoquinolines, dihydrobenzo-1,4-oxazines, and dihydrobenzo-3,1-oxazines using our developed mixed double-Michael strategy [31].

Focusing on oxazolidines, here we report the synthesis of a library of 60 distinct oxazolidines through our mixed double-Michael strategy. Based on previously reported mixed double-Michael reactions [30,31], we knew that oxazolidines could be prepared from amino acid–derived pro-nucleophiles and electron-deficient acetylenes using DPPP as the catalyst. With such precedents in mind, here we expanded the reaction scope of the mixed double-Michael reaction to incorporate various oxygen-and-nitrogen-containing pro-nucleophiles and electron-deficient acetylenes. Under the reported conditions, we obtained the various oxazolidine derivatives rapidly and with high efficiency, enabling their future application in biological assays.

Oxazolidine, a five-membered-ring heterocycle containing both oxygen and nitrogen atoms, appears in many natural products that possess important biological activities. Quinocarcin and tetrazomine are two examples of the many naturally occurring oxazolidine-containing molecules displaying useful pharmacological activities (e.g., antitumor, cytotoxic, anti-inflammatory, and analgesic properties) [38,39]. Furthermore, enantiomerically pure oxazolidines can be used as chiral auxiliaries to induce asymmetry in organic reactions [40,41,42,43,44,45].

## 2. Results and Discussion

### 2.1. Preparation of Pro-Nucleophiles

The β-amino alcohol pro-nucleophiles that we used in the mixed double-Michael reactions were derived from natural l-amino acids and racemic cyclohexene oxide. We prepared the pro-nucleophiles derived from l-leucine, l-alanine, l-phenylalanine, and l-serine efficiently according to protocols described by Moberg and Craig [46]; first, we protected the amino groups of l-leucine, l-alanine, l-phenylalanine, and l-serine with *p*-toluenesulfonyl (tosyl) chloride and then we reduced their carboxylic acid units using lithium aluminum hydride [47,48]. We prepared the 2-aminocyclohexanol–derived pro-nucleophile **1e** from racemic cyclohexene oxide in three steps: opening of the epoxide with sodium azide, reduction of the azido group with palladium on charcoal [49,50], and then protection of the amino group with tosyl chloride [51].

### 2.2. Preparation of Electron-Deficient Acetylenes

Table 1 lists the electron-deficient acetylenes that we prepared from corresponding aldehydes. Although the syntheses of the acetylenes **2a**, **2c**, and **2e** have been reported previously, the acetylenes **2b**, **2d**, and **2f** were unknown. Employing the protocol established by Oyelere and Calieno [52,53], we obtained each of these propargyl ketones in good yield after: (i) treating the pertinent aldehyde with ethynylmagnesium bromide at 0 °C, slowly warming to ambient temperature, and then working-up the mixture after 4 h and (ii) oxidizing the resulting propargyl alcohol, without further purification, using Jones reagent.

### 2.3. Synthesis of an Oxazolidine Library

With all the pro-nucleophiles and acetylenes at hand, we rapidly synthesized the desired chemical library of 60 distinct oxazolidines using DPPP as the catalyst (Table 2, Table 3 and Table 4). The oxazolidine library contained a diverse array of functional groups and provided several potential probes for examining various biological mechanisms.

The pro-nucleophiles **1a**–**c** generated their desired oxazolidines in good to high yields, revealing that the reactions had good tolerance for various alkyl groups on the pro-nucleophiles (Table 2, entries 1–33). In contrast, the pro-nucleophile **1d** provided its products in low to moderate yields, suggesting that the additional free hydroxyl functionality impeded the mixed double-Michael reactions (Table 2, entries 34–44). The yields of oxazolidines were even lower when we used **1e** as the pro-nucleophile (Table 3), presumably because of steric hindrance about the secondary alcohol pro-nucleophile.

We tested various activated acetylenes to evaluate their reactivities in the mixed double-Michael reactions. Alkyl propiolates afforded their desired oxazolidines in good to high yields (Table 1, entries 1–3, 12–14, 23–25, and 34–36). In contrast, the use of phenyl propiolate provided drastically lower yields of its oxazolidines (Table 1, entries 4, 15, 26, and 37), possibly because it was prone to hydrolysis under the reaction conditions.

Butynone and aromatic propargyl ketones afforded their oxazolidines in good yields from a range of pro-nucleophiles. Halogens and electron-donating groups on the benzene rings of the aromatic propargyl ketones were compatible with the mixed double-Michael reactions. Naphthyl and 2-thienyl groups were also tolerated in the reaction. Aside from using carbonyl moieties as electron withdrawing groups, we also employed an aromatic sulfone unit to activate the acetylene; tosyl acetylene provided its oxazolidines in good yields from the mixed double-Michael reactions (Table 4).

## 3. Experimental 

### 3.1. General 

All reactions were performed in flamed-dried or oven-dried round-bottom flasks, Schlenk flasks, or two-neck flasks. A glass water condenser, fitted with a rubber septum, was attached to each flask. All reactions were performed under a positive pressure of argon. A syringe pump and stainless-steel needles were used to inject the acetylene derivatives into the refluxing reaction mixtures. Reactions were monitored through thin-layer chromatography (TLC) on 0.25-mm SiliCycle silica gel plates. Plates were visualized under UV light or through *p*-anisaldehyde or potassium permanganate staining followed by heating (<1 min) with a heat gun. Flash column chromatography (FCC) was performed using SiliCycle Silica-P Flash silica gel (60 Å pore size, 40–63 µm). Organic solutions were concentrated using rotary evaporators.

### 3.2. Materials and Reagents

Reagents were used as received from commercial sources. Methyl propiolate and ethyl propiolate were purchased from TCI America. Tosyl acetylene and 3-butyn-2-one were purchased from Aldrich. Acetonitrile and dichloromethane were distilled from calcium hydride under a positive pressure of argon. Tetrahydrofuran was distilled from sodium and benzophenone under a positive pressure of argon.

### 3.3. Instrumentation

IR spectra were recorded using a Thermo Nicolet Avatar 370 FT-IR spectrometer. NMR spectra were recorded using Bruker ARX-400 instrument, calibrated to signals from the solvent as an internal reference [7.26 (residual CHCl_3_) and 77.00 (CDCl_3_) ppm for ¹H- and ¹³C-NMR spectra, respectively]. Data for ^1^H NMR spectra are reported as follows: chemical shift (δ, ppm), multiplicity, coupling constant (Hz), and integration. Data for ^13^C-NMR spectra are reported in terms of chemical shift. The following abbreviations are used to denote the multiplicities: s = singlet; d = doublet; t = triplet; q = quartet; m = multiplet. Mass spectra of the samples were recorded using a Waters LCT Premier XE time-of-flight instrument controlled by MassLynx 4.1 software. Samples were infused through direct loop injection from a Waters Acquity UPLC into the multi-mode ionization source. The lock mass standard for accurate mass determination was leucine enkephalin (Sigma L9133). An Agilent Technologies 5975 inert XL mass-selective detector GCMS was also used.

### 3.4. Synthesis of Electron-Deficient Acetylenes

*1-Phenylprop-2-yn-1-one* (**2a**) was prepared in 83% yield using the protocol described by Oyelere and Calieno [52,53]. Spectral data matched those reported in the literature [53].

*1-(3,4-Dichlorophenyl)prop-2-yn-1-one* (**2b**) was prepared in 76% yield using the protocol described by Oyelere and Calieno [52,53]; yellow solid; IR (CH_2_Cl_2_) *ν*_max_ 3219.3, 2093.63, 1639.0, 1579.0, 1232.4, 750.1 cm^–1^; ^1^H-NMR (400 MHz, CDCl_3_) *δ* 8.23 (s, 1H), 7.97–8.00 (m, 1H), 7.58–7.61 (m, 1H), 3.51 (s, 1H); ^13^C-NMR (100 MHz, CDCl_3_) *δ* 174.9, 139.4, 135.7, 133.5, 130.9, 128.4, 81.9, 79.5, 77.2; GCMS (EI+) calcd for [C_9_H_4_Cl_2_O]: *m*/*z* 199.0, found 199.0.

*1-(3,4-Dimethoxyphenyl)prop-2-yn-1-one* (**2c**): 89% yield; spectral data matched those reported in the literature [54].

*1-(4-Fluorophenyl)prop-2-yn-1-one* (**2d**) was prepared in 76% yield using the protocol described by Oyelere and Calieno [52,53]; brown solid; IR (CH_2_Cl_2_) *ν*_max_ 3211.7, 2093.45, 1651.4, 1598.3, 1255.5, 1155.4, 754.6 cm^–1^; ^1^H-NMR (400 MHz, CDCl_3_) *δ* 8.20 (d, *J* = 4.0 Hz, 2H), 7.18 (d, *J* = 4.0 Hz, 2H), 3.45 (s, 1H); ^13^C-NMR (100 MHz, CDCl_3_) *δ* 175.7, 168.0, 165.4, 132.5, 116.1, 115.9, 81.0, 80.0, 77.2; GCMS (EI+) calcd for [C_9_H_5_FO]: *m*/*z* 148.1, found 148.1.

*1-(Naphthalen-1-yl)prop-2-yn-1-one* (**2e**): 67% yield; spectral data matched those reported in the literature [55].

*1-(Thien-2-yl)prop-2-yn-1-one* (**2f**) was prepared in 75% yield using the protocol described by Oyelere and Calieno [52,53]; brown solid; IR (CH_2_Cl_2_) *ν*_max_ 3239.2, 2093.3, 1619.1, 1409.8, 1279.2, 734.4 cm^–1^; ^1^H-NMR (400 MHz, CDCl_3_) *δ* 7.96 (d, *J* = 4.0 Hz, 1H), 7.74 (d, *J* = 4.0 Hz, 1H), 7.16 (t, *J* = 4.0 Hz, 1H), 3.37 (s, 1H); ^13^C-NMR (100 MHz, CDCl_3_) *δ* 169.1, 144.1, 136.2, 129.1, 128.5, 79.4, 77.3; GCMS (EI+) calcd for [C_7_H_4_OS]: *m*/*z* 136.2, found 136.1.

*Phenyl propiolate* was prepared in 90% yield according to the protocol described by Ramachandran; spectral data matched those reported in the literature [56].

*Benzyl propiolate* was prepared in 90% yield according to the protocol described by Ramachandran; spectral data matched those reported in the literature [57].

### 3.5. General Procedure for Mixed Double-Michael Reaction

The *N*-tosylamido alcohol pro-nucleophile (1 mmol), DPPP (84.2 mg, 0.2 mmol), and CH_3_CN (5 mL) were placed in a dried two-neck round-bottom flask (25 mL) equipped with a magnetic stirrer bar. The mixture was brought to reflux in an oil bath (external temperature: 90 °C). A solution of the electron-deficient acetylene (1.2 mmol) in CH_3_CN (1 mL) was added into the flask over 1 h using a syringe pump. [In the case of ethynyl *p*-tolyl sulfone, the acetylene (1.2 mmol) was added to the flask over 2 h via syringe pump.] The reaction mixture was stirred overnight under reflux, under a positive pressure of argon, monitoring with TLC (20% EtOAc in hexanes). The resulting reaction mixture was concentrated in vacuo and purified by FCC on silica gel (20% EtOAc in hexanes) to yield the cyclized product.

*Methyl 2-[(2S,4S)-4-isopropyl-3-tosyloxazolidin-2-yl]acetate* (**3a**). 59% yield; white solid; IR (CH_2_Cl_2_) *ν*_max_ 2960.7, 1741.9, 1598.1, 1351.2, 1164.7 cm^–1^; ^1^H-NMR (400 MHz, CDCl_3_) *δ* 7.68 (d, *J* = 8.4 Hz, 2H), 7.29 (d, *J* = 8.0 Hz, 2H), 5.23 (dd, *J* = 8.8, 3.2 Hz, 1H), 3.69 (dd, *J* = 9.2, 2.4 Hz, 1H), 3.64 (s, 3H), 3.35 (ddd, *J* = 7.9, 6.1, 2.1 Hz, 1H), 3.00–3.07 (m, 2H), 2.62 (dd, *J* = 15.8, 8.6 Hz, 1H), 2.36 (s, 3H), 1.78–1.86 (m, 1H), 0.95 (d, *J* = 6.8 Hz, 3H), 0.87 (d, *J* = 6.8 Hz, 3H); ^13^C-NMR (100 MHz, CDCl_3_) *δ* 169.9, 144.4, 133.7, 130.0, 127.9, 88.8, 67.8, 64.9, 51.8, 41.8, 31.3, 21.5, 19.5, 18.4; HRMS (ESI+) calcd for [C_16_H_23_NO_5_S]^+^: *m*/*z* 342.1370, found 342.1364.

*Ethyl 2-[(2S,4S)-4-isopropyl-3-tosyloxazolidin-2-yl]acetate* (**3b**). 75% yield; yellow oil; IR (CH_2_Cl_2_) *ν*_max_ 2964.4, 1736.0, 1598.0, 1351.6, 1165.3 cm^–1^; ^1^H-NMR (400 MHz, CDCl_3_) *δ* 7.65 (d, *J* = 8.4 Hz, 2H), 7.25 (d, *J* = 8.0 Hz, 2H), 5.21 (dd, *J* = 8.8, 3.2 Hz, 1H), 4.07 (q, *J* = 7.2 Hz, 2H), 3.66 (dd, *J* = 9.0, 2.2 Hz, 1H), 3.32 (ddd, *J* = 7.7, 6.1, 1.9 Hz, 1H), 2.96–3.04 (m, 2H), 2.58 (dd, *J* = 15.8, 8.6 Hz, 1H), 2.32 (s, 3H), 1.75–1.84 (m, 1H), 1.17 (t, *J* = 7.0 Hz, 3H), 0.92 (d, *J* = 6.8 Hz, 3H), 0.84 (d, *J* = 6.8 Hz, 3H); ^13^C-NMR (100 MHz, CDCl_3_) *δ* 169.5, 144.3, 133.7, 130.0, 127.9, 88.9, 67.8, 64.8, 60.7, 42.0, 31.3, 21.4, 19.5, 18.4, 14.1; HRMS (ESI+) calcd for [C_17_H_25_NO_5_S]^+^: *m*/*z* 356.1526, found 356.1518.

*Benzyl 2-[(2S,4S)-4-isopropyl-3-tosyloxazolidin-2-yl]acetate* **(3c**). 63% yield; yellow oil; IR (CH_2_Cl_2_) *ν*_max_ 3031.4, 2962.6, 1738.7, 1597.8, 1351.2, 1164.7 cm^–1^; ^1^H-NMR (400 MHz, CDCl_3_) *δ* 7.73 (d, *J* = 8.0 Hz, 2H), 7.30–7.36 (m, 7H), 5.35 (dd, *J* = 8.6, 3.0 Hz, 1H), 5.15 (s, 2H), 3.75 (dd, *J* = 9.0, 2.2 Hz, 1H), 3.42 (ddd, *J* = 7.7, 5.9, 2.1 Hz, 1H), 3.09–3.17 (m, 2H), 2.75 (dd, *J* = 15.8, 8.6 Hz, 1H), 2.40 (s, 3H), 1.84–1.91 (m, 1H), 1.01 (d, *J* = 6.8 Hz, 3H), 0.92 (d, *J* = 6.8 Hz, 3H); ^13^C-NMR (100 MHz, CDCl_3_) *δ* 169.5, 144.4, 135.7, 133.8, 130.1, 130.0, 128.6, 128.3, 128.0, 88.9, 67.9, 66.6, 65.0, 42.0, 31.4, 21.6, 19.6, 18.5. HRMS (ESI+) calcd for [C_22_H_27_NO_5_S]^+^: *m*/*z* 418.1683, found 418.1671.

*Phenyl 2-[(2S,4S)-4-isopropyl-3-tosyloxazolidin-2-yl]acetate* (**3d**). 22% yield; yellow oil; IR (CH_2_Cl_2_) *ν*_max_ 3065.2, 2963.8, 1758.9, 1594.3, 1351.3, 1306.6, 1164.5 cm^–1^; ^1^H-NMR (400 MHz, CDCl_3_) *δ* 7.77 (d, *J* = 8.4 Hz, 2H), 7.34–7.37 (m, 4H), 7.21–7.25 (m, 1H), 7.12 (d, *J* = 1.2 Hz, 2H), 5.42 (dd, *J* = 8.2, 3.4 Hz, 1H), 3.82 (dd, *J* = 9.2, 2.4 Hz, 1H), 3.44 (ddd, *J* = 7.8, 6.0, 2.0 Hz, 1H), 3.34 (dd, *J* = 15.8, 3.4 Hz, 1H), 3.16 (dd, *J* = 9.2, 6.4 Hz, 1H), 2.93 (dd, *J* = 15.8, 8.2 Hz, 1H), 2.43 (s, 3H), 1.93–1.99 (m, 1H), 1.05 (d, *J* = 6.8 Hz, 3H), 0.98 (d, *J* = 6.8 Hz, 3H); ^13^C-NMR (100 MHz, CDCl_3_) *δ* 168.2, 150.5, 144.5, 133.7, 130.1 , 129.4, 128.1, 126.0, 121.6, 88.9, 67.9, 65.0, 42.2, 31.4, 21.6, 19.6, 18.5; HRMS (ESI+) calcd for [C_21_H_25_NO_5_S]^+^: *m*/*z* 404.1526, found 404.1510.

*1-[(2S,4S)-4-Isopropyl-3-tosyloxazolidin-2-yl]propan-2-one* (**3e**). 53% yield; light yellow oil; IR (CH_2_Cl_2_) *ν*_max_ 2963.3, 1719.4, 1597.9, 1348.5, 1164.1 cm^–1^; ^1^H-NMR (400 MHz, CDCl_3_) *δ* 7.65 (d, *J* = 8.4 Hz, 2H), 7.27 (d, *J* = 8.4 Hz, 2H), 5.19 (dd, *J* = 8.4, 2.4 Hz, 1H), 3.66 (dd, *J* = 9.0, 1.8 Hz, 1H), 3.31 (ddd, *J* = 7.6, 6.0, 1.6 Hz, 1H), 3.12 (dd, *J* = 17.0, 2.6 Hz, 1H), 2.99 (dd, *J* = 9.0, 6.2 Hz, 1H), 2.78 (dd, *J* = 16.8, 8.4 Hz, 1H), 2.35 (s, 3H), 2.13, (s, 3H), 1.76–1.85 (m, 1H), 0.94 (d, *J* = 6.8 Hz, 3H), 0.86 (d, *J* = 6.8 Hz, 3H); ^13^C-NMR (100 MHz, CDCl_3_) *δ* 205.1, 144.3, 133.6, 130.0, 127.9, 88.3, 67,8, 64.7, 50.3, 31.4, 30.7, 21.5, 19.5, 18.4; HRMS (ESI+) calcd for [C_16_H_23_NO_4_S]^+^: *m*/*z* 326.1421, found 326.1416.

*2-[(2S,4S)-4-Isopropyl-3-tosyloxazolidin-2-yl]-1-phenylethanone* (**3f**). 55% yield; yellow solid; IR (CH_2_Cl_2_) *ν*_max_ 3062.5, 2963.3, 1686.1, 1597.3, 1349.4, 1164.5 cm^–1^; ^1^H-NMR (400 MHz, CDCl_3_) *δ* 7.95 (d, *J* = 6.8 Hz, 2H), 7.72 (d, *J* = 8.0 Hz, 2H), 7.41–7.53 (m, 3H), 7.31 (d, *J* = 12.0 Hz, 2H), 5.45 (dd, *J* = 8.8, 2.0 Hz, 1H), 3.74 (dq, *J* = 12.8, 2.0 Hz, 2H), 3.35–3.43 (m, 2H), 3.06 (dd, *J* = 8.8, 6.0 Hz, 1H), 2.37 (s, 3H), 1.86–1.95 (m, 1H), 1.02 (d, *J* = 7.2 Hz, 3H), 0.94 (d, *J* = 6.8 Hz, 3H); ^13^C-NMR (100 MHz, CDCl_3_) *δ* 196.6, 144.4, 136.6, 133.6, 133.5, 130.1, 128.7, 128.2, 128.0, 89.0, 67.9, 64.8, 45.9, 31.5, 21.5, 19.6, 18.5; HRMS (ESI+) calcd for [C_21_H_25_NO_4_S]^+^: *m*/*z* 388.1577, found 388.1568.

*1-(3,4-Dichlorophenyl)-2-[(2S,4S)-4-isopropyl-3-tosyloxazolidin-2-yl]ethanone* (**3g**). 28% yield; yellow solid; IR (CH_2_Cl_2_) *ν*_max_ 3064.1, 2963.2, 1691.3, 1584.2, 1350.0, 1164.8 cm^–1^; ^1^H-NMR (400 MHz, CDCl_3_) *δ* 8.02 (d, *J* = 2.0 Hz, 2H), 7.78 (dd, *J* = 8.4, 2.0 Hz, 1H), 7.72 (d, *J* = 8.4 Hz, 2H), 7.53 (d, *J* = 8.4 Hz, 1H), 7.33 (d, *J* = 8.4 Hz, 2H), 5.38 (dd, *J* = 8.4, 2.0 Hz, 1H), 3.76 (dd, *J* = 9.2, 1.6 Hz, 1H), 3.71 (dd, *J* = 16.6, 2.2 Hz, 1H), 3.39 (ddd, *J* = 7.6, 6.0, 1.6 Hz, 1H), 3.06 (dd, *J* = 9.0, 6.2 Hz, 1H), 3.06 (dd, *J* = 9.0, 6.2 Hz, 1H), 2.41 (s, 3H), 1.85–1.92 (m, 1H), 1.02 (d, *J* = 7.2 Hz, 3H), 0.94 (d, *J* = 6.8 Hz, 3H); ^13^C-NMR (100 MHz, CDCl_3_) *δ* 194.5, 144.5, 138.0, 136.1, 133.5, 133.4, 130.8, 130.2, 130.1, 128.0, 127.4, 88.7, 67.9, 64.8, 46.0, 31.5, 21.6, 19.6, 18.5; HRMS (ESI+) calcd for [C_21_H_23_Cl_2_NO_4_S]^+^: *m*/*z* 456.0798, found 456.0756.

*1-(3,4-Dimethoxyphenyl)-2-[(2S,4S)-4-isopropyl-3-tosyloxazolidin-2-yl]ethanone* (**3h**). 58% yield; yellow oil; IR (CH_2_Cl_2_) *ν*_max_ 3068.2, 2962.9, 1673.5, 1595.7, 1347.3, 1164.0 cm^–1^; ^1^H-NMR (400 MHz, CDCl_3_) *δ* 7.66 (d, *J* = 8.4 Hz, 2H), 7.54 (dd, *J* = 8.4, 2.0 Hz, 1H), 7.25 (d, *J* = 8.4 Hz, 2H), 5.36 (dd, *J* = 8.8, 2.0 Hz, 1H), 3.84 (s, 3H), 3.83 (s, 3H), 3.61–3.70 (m, 2H), 3.33 (ddd, *J* = 7.5, 6.1, 1.7 Hz, 1H), 3.24 (dd, *J* = 16.4, 8.8 Hz, 1H), 2.32 (s, 3H), 1.80–1.89 (m, 1H), 0.95 (d, *J* = 7.2 Hz, 3H), 0.87 (d, *J* = 6.8 Hz, 3H); ^13^C-NMR (100 MHz, CDCl_3_) *δ* 195.1, 153.6, 149.0, 144.3, 133.6, 130.0, 129.8, 128.0, 123.2, 110.2, 89.2, 67.8, 64.7, 56.0, 55.9, 45.4, 31.4, 21.4, 19.6, 18.4; HRMS (ESI+) calcd for [C_23_H_29_NO_6_S]^+^: *m*/*z* 448.1788, found 448.1790.

*1-(4-Fluorophenyl)-2-[(2S,4S)-4-isopropyl-3-tosyloxazolidin-2-yl]ethanone* (**3i**). 63% yield; brown oil; IR (CH_2_Cl_2_) *ν*_max_ 3068.8, 2963.8, 1686.0, 1597.3, 1349.6, 1164.7 cm^–1^; ^1^H-NMR (400 MHz, CDCl_3_) *δ* 7.97 (t, *J* = 6.8 Hz, 2H), 7.70 (d, *J* = 7.6 Hz, 2H), 7.30 (d, *J* = 8.0 Hz, 2H), 7.09 (t, *J* = 8.0 Hz, 2H), 5.40 (dd, *J* = 8.6, 1.8 Hz, 1H), 3.67–3.74 (m, 2H), 3.29–3.40 (m, 2H), 3.04 (dd, *J* = 8.8, 6.4 Hz, 1H), 2.37 (s, 3H), 1.84–1.92 (m, 1H), 1.00 (d, *J* = 6.8 Hz, 3H), 0.92 (d, *J* = 6.8 Hz, 3H); ^13^C-NMR (100 MHz, CDCl_3_) *δ* 195.0, 167.2, 164.6, 144.4, 133.6, 133.1, 131.0, 130.1, 130.0, 128.0, 115.8, 115.6, 89.0, 67.9, 64.8, 45.8, 31.5, 21.5, 19.6, 18.5; HRMS (ESI+) calcd for [C_21_H_24_FNO_4_S]^+^: *m*/*z* 406.1483, found 406.1477.

*2-[(2S,4S)-4-Isopropyl-3-tosyloxazolidin-2-yl]-1-(naphth-1-yl)ethanone* (**3j**). 70% yield; yellow oil; IR (CH_2_Cl_2_) *ν*_max_ 3051.1, 2963.2, 1680.3, 1595.9, 1348.4, 1164.0 cm^–1^; ^1^H-NMR (400 MHz, CDCl_3_) *δ* 8.72 (d, *J* = 8.4 Hz, 1H), 7.96–7.98 (m, 2H), 7.84 (d, *J* = 8.0 Hz, 1H), 7.77 (d, *J* = 8.0 Hz, 2H), 7.48–7.59 (m, 3H), 7.27 (d, *J* = 11.6 Hz, 2H), 5.55 (dd, *J* = 8.8, 2.4 Hz, 1H), 3.92 (dd, *J* = 16.6, 2.2 Hz, 1H), 3.79 (dd, *J* = 9.0, 1.8 Hz, 1H), 3.45 (ddd, *J* = 13.5, 6.7, 4.5 Hz, 2H), 3.10 (dd, *J* = 9.0, 6.2 Hz, 1H), 2.37 (s, 3H), 1.90–1.98 (m, 1H), 1.05 (d, *J* = 6.8 Hz, 3H), 0.96 (d, *J* = 6.4 Hz, 3H); ^13^C-NMR (100 MHz, CDCl_3_) *δ* 200.3, 144.4, 135.0, 134.0, 133.7, 133.3, 130.2, 130.1, 128.5, 128.1, 126.6, 126.0, 124.4, 89.4, 68.0, 64.9, 49.1, 31.6, 21.5, 19.7, 18.6; HRMS (ESI+) calcd for [C_25_H_27_NO_4_S]^+^: *m*/*z* 438.1734, found 438.1730. 

*2-[(2S,4S)-4-Isopropyl-3-tosyloxazolidin-2-yl]-1-(thien-2-yl)ethanone* (**3k**). 37% yield; brown solid; IR (CH_2_Cl_2_) *ν*_max_ 3102.5, 2963.1, 1660.1, 1597.6, 1348.8, 1164.0 cm^–1^; ^1^H-NMR (400 MHz, CDCl_3_) *δ* 7.77 (d, *J* = 1.2 Hz, 1H), 7.72 (d, *J* = 8.4 Hz, 2H), 7.64 (dd, *J* = 5.0, 1.0 Hz, 1H), 7.32 (d, *J* = 8.0 Hz, 2H), 7.12 (dd, *J* = 5.0, 3.8 Hz, 1H), 5.39 (dd, *J* = 8.4, 2.4 Hz, 1H), 3.75 (dd, *J* = 9.2, 2.0 Hz, 1H), 3.64 (dd, *J* = 15.8, 2.2 Hz, 1H), 3.39 (ddd, *J* = 7.6, 6.0, 1.8 Hz, 1H), 3.30 (dd, *J* = 16.0, 8.4 Hz, 1H), 3.06 (dd, *J* = 8.8, 6.0 Hz, 1H), 2.40 (s, 3H), 1.86–1.95 (m, 1H), 1.01 (d, *J* = 6.8 Hz, 3H), 0.94 (d, *J* = 6.8 Hz, 3H); ^13^C-NMR (100 MHz, CDCl_3_) *δ* 189.1, 144.4, 144.1, 134.4, 133.6, 132.8, 130.1, 128.3, 128.0, 89.0, 67.9, 64.8, 46.5, 31.5, 21.6, 19.6, 18.5; HRMS (ESI+) calcd for [C_19_H_23_NO_4_S_2_]^+^: *m*/*z* 394.1141, found 394.1152.

*Methyl 2-[(2S,4S)-4-methyl-3-tosyloxazolidin-2-yl]acetate* (**4a**). 68% yield; white solid; IR (CH_2_Cl_2_) *ν*_max_ 2983.0, 1739.9, 1598.2, 1350.8, 1164.8 cm^–1^; ^1^H-NMR (400 MHz, CDCl_3_) *δ* 7.69 (d, *J* = 1.6 Hz, 2H), 7.30 (d, *J* = 8.0 Hz, 2H), 5.29 (dd, *J* = 8.8, 2.8 Hz, 1H), 3.65–3.70 (m, 4H), 3.52 (dd, *J* = 9.0, 4.2 Hz, 1H), 3.41 (dd, *J* = 8.8, 6.0 Hz, 1H), 3.04 (dd, *J* = 16.0, 3.2 Hz, 1H), 2.69 (dd, *J* = 15.6, 8.8 Hz, 1H), 2.38 (s, 3H), 1.29 (d, *J* = 6.4 Hz, 3H); ^13^C-NMR (100 MHz, CDCl_3_) *δ* 169.9, 144.4, 133.6, 130.0, 127.8, 127.1, 88.9, 71.8, 55.1, 51.9, 51.8, 42.1, 21.5, 20.9; HRMS (ESI+) calcd for [C_14_H_19_NO_5_S]^+^: *m*/*z* 314.1057, found 314.1050.

*Ethyl 2-[(2S,4S)-4-methyl-3-tosyloxazolidin-2-yl]acetate* (**4b**). 68% yield; white solid; IR (CH_2_Cl_2_) *ν*_max_ 2982.7, 1736.4, 1598.3, 1351.1, 1164.9 cm^–1^; ^1^H-NMR (400 MHz, CDCl_3_) *δ* 7.65 (d, *J* = 8.4 Hz, 2H), 7.26 (d, *J* = 8.0 Hz, 2H), 5.25 (dd, *J* = 8.6, 3.0 Hz, 1H), 4.08 (q, *J* = 7.1 Hz, 2H), 3.65 (ddd, *J* = 8.3, 4.3, 2.1 Hz, 1H), 3.48 (dd, *J* = 9.0, 4.2 Hz, 1H), 3.37 (dd, *J* = 8.8, 6.0 Hz, 1H), 2.98 (dd, *J* = 15.8, 3.0 Hz, 1H), 2.63 (dd, *J* = 15.6. 8.8 Hz, 1H), 2.33 (s, 3H), 1.24 (d, *J* = 6.4 Hz, 3H), 1.18 (t, *J* = 7.2 Hz, 3H); ^13^C-NMR (100 MHz, CDCl_3_) *δ* 169.4, 144.3, 133.6, 130.0, 127.8, 127.1, 88.9, 71.8, 60.7, 55.0, 42.2, 21.4, 20.8, 14.1; HRMS (ESI+) calcd for [C_15_H_21_NO_5_S]^+^: *m*/*z* 328.1213, found 328.1207.

*Benzyl 2-[(2S,4S)-4-methyl-3-tosyloxazolidin-2-yl]acetate* (**4c**). 64% yield; yellow oil; IR (CH_2_Cl_2_) *ν*_max_ 3064.8, 2979.9, 1738.1, 1598.1, 1351.1, 1165.1 cm^–1^; ^1^H-NMR (400 MHz, CDCl_3_) *δ* 7.73 (d, *J* = 8.4 Hz, 2H), 7.28–7.36 (m, 6H), 5.38 (dd, *J* = 8.6, 3.0 Hz, 1H), 5.16 (s, 2H), 3.73 (ddd, *J* = 8.5, 4.1, 2.1 Hz, 1H), 3.55 (dd, *J* = 8.8, 4.0 Hz, 1H), 3.46 (dd, *J* = 9.0, 6.2 Hz, 1H), 3.14 (dd, *J* = 15.8, 3.0 Hz, 1H), 2.79 (dd, *J* = 15.8, 8.6 Hz, 1H), 2.41 (s, 3H), 1.32 (d, *J* = 6.4 Hz, 3H); ^13^C-NMR (100 MHz, CDCl_3_) *δ* 169.4, 144.4, 135.7, 133.6, 130.1, 130.0, 128.6, 128.5, 128.3, 127.9, 127.2, 89.0, 71.9, 66.6, 55.2, 42.3, 21.6, 20.9; HRMS (ESI+) calcd for [C_20_H_23_NO_5_S]^+^: *m*/*z* 390.1317, found 390.1346.

*Phenyl 2-[(2S,4S)-4-methyl-3-tosyloxazolidin-2-yl]acetate* (**4d**). 14% yield; yellow oil; IR (CH_2_Cl_2_) *ν*_max_ 3068.2, 2980.3, 1758.4, 1593.7, 1350.8, 1164.3 cm^–1^; ^1^H-NMR (400 MHz, CDCl_3_) *δ* 7.77 (d, *J* = 8.4 Hz, 2H), 7.34–7.40 (m, 5H), 7.23–7.25 (m, 1H), 7.12 (d, *J* = 1.2 Hz, 2H), 5.45 (dd, *J* = 8.2, 3.4 Hz, 1H), 3.76 (ddd, *J* = 8.4, 4.4, 2.0 Hz, 1H), 3.64 (dd, *J* = 8.8, 4.0 Hz, 1H), 3.52 (dd, *J* = 8.8, 6.4 Hz, 1H), 3.34 (dd, *J* = 15.8, 3.4 Hz, 1H), 2.99 (dd, *J* = 15.8, 8.2 Hz, 1H), 2.44 (s, 3H), 1.39 (d, *J* = 6.4 Hz, 3H); ^13^C-NMR (100 MHz, CDCl_3_) *δ* 168.0, 150.5, 144.4, 133.6, 130.1, 129.4, 129.1, 127.9, 126.0, 121.6, 88.9, 72.0, 55.2, 42.5, 21.6, 21.0; HRMS (ESI+) calcd for [C_19_H_21_NO_5_S]^+^: *m*/*z* 376.1213, found 376.1204.

*1-[(2S,4S)-4-Methyl-3-tosyloxazolidin-2-yl]propan-2-one* (**4e**). 68% yield; yellow oil; IR (CH_2_Cl_2_) *ν*_max_ 2980.2, 1715.8, 1598.2, 1348.8, 1164.6 cm^–1^; ^1^H-NMR (400 MHz, CDCl_3_) *δ* 7.66 (d, *J* = 8.4 Hz, 2H), 7.28 (d, *J* = 8.0 Hz, 2H), 5.23 (dd, *J* = 8.4, 2.4 Hz, 1H), 3.65 (ddd, *J* = 7.8, 4.6, 1.8 Hz, 1H), 3.47 (dd, *J* = 8.8, 3.6 Hz, 1H), 3.32 (dd, *J* = 9.0, 6.2 Hz, 1H), 3.11 (dd, *J* = 16.6, 2.6 Hz, 1H), 2.84 (dd, *J* = 16.4. 8.4 Hz, 1H), 2.36 (s, 3H), 2.14 (s, 3H), 1.26 (d, *J* = 6.8 Hz, 3H); ^13^C-NMR (100 MHz, CDCl_3_) *δ* 205.1, 144.3, 133.5, 130.0, 127.8, 126.9, 88.4, 71.8, 54.9, 50.6, 30.8, 21.5, 21.0; HRMS (ESI+) calcd for [C_14_H_19_NO_4_S]^+^: *m*/*z* 298.1108, found 298.1128.

*2-[(2S,4S)-4-Methyl-3-tosyloxazolidin-2-yl]-1-phenylethanone* (**4f**). 57% yield; white solid; IR (CH_2_Cl_2_) *ν*_max_ 3062.3, 2980.2, 1686.7, 1597.3, 1348.9, 1164.5 cm^–1^; ^1^H-NMR (400 MHz, CDCl_3_) *δ* 7.97 (d, *J* = 7.2 Hz, 2H), 7.73 (d, *J* = 8.0 Hz, 2H), 7.53–7.74 (m, 1H), 7.45 (t, *J* = 7.6 Hz, 2H), 7.31 (d, *J* = 8.0 Hz, 2H), 5.50 (dd, *J* = 8.8, 2.0 Hz, 1H), 3.71–3.75 (m, 2H), 3.55 (dd, *J* = 8.8, 3.6 Hz, 1H), 3.37–3.45 (m, 2H), 2.39 (s, 3H), 1.35 (d, *J* = 6.4 Hz, 3H); ^13^C-NMR (100 MHz, CDCl_3_) *δ* 196.6, 144.4, 136.6, 133.5, 130.1, 130.0, 129.6, 128.7, 128.3, 127.9, 89.0, 71.9, 55.0, 46.3, 21.6, 21.1; HRMS (ESI+) calcd for [C_19_H_21_NO_4_S]^+^: *m*/*z* 360.1264, found 360.1260.

*1-(3,4-Dichlorophenyl)-2-[(2S,4S)-4-methyl-3-tosyloxazolidin-2-yl]ethanone* (**4g**). 41% yield; brown oil; IR (CH_2_Cl_2_) *ν*_max_ 3065.8, 2980.5, 1692.7, 1584.3, 1349.3, 1164.8 cm^–1^; ^1^H-NMR (400 MHz, CDCl_3_) *δ* 8.03 (d, *J* = 2.0 Hz, 1H), 7.79 (dd, *J* = 8.4, 2.0 Hz, 1H), 7.71 (d, *J* = 8.4 Hz, 2H), 7.32 (d, *J* = 8.0 Hz, 2H), 5.42 (dd, *J* = 8.4, 2.0 Hz, 1H), 3.67–3.74 (m, 2H), 3.55 (dd, *J* = 9.0, 3.4 Hz, 1H), 3.32–3.40 (m, 2H), 2.40 (s, 3H), 1.34 (d, *J* = 6.4 Hz, 3H); ^13^C-NMR (100 MHz, CDCl_3_) *δ* 194.4, 144.5, 138.4, 137.0, 134.0, 133.4, 131.1, 130.8, 130.0, 128.3, 127.4, 88.8, 72.0, 55.0, 46.3, 21.6, 21.1; HRMS (ESI+) calcd for [C_19_H_19_Cl_2_NO_4_S]^+^: *m*/*z* 428.0485, found 428.0476.

*1-(3,4-Dimethoxyphenyl)-2-[(2S,4S)-4-methyl-3-tosyloxazolidin-2-yl]ethanone* (**4h**). 90% yield; yellow oil; IR (CH_2_Cl_2_) *ν*_max_ 3064.1, 2974.2, 1672.7, 1595.4, 1346.4, 1163.4 cm^–1^; ^1^H-NMR (400 MHz, CDCl_3_) *δ* 7.64 (d, *J* = 8.0 Hz, 2H), 7.53 (dd, *J* = 8.4, 2.0 Hz, 1H), 7.43 (d, *J* = 2.0 Hz, 1H), 7.22 (d, *J* = 8.0 Hz, 2H), 6.81 (d, *J* = 8.4 Hz, 1H), 5.40 (dd, *J* = 8.8, 2.0 Hz, 1H), 3.78–3.81 (m, 7H), 3.62 (ddd, *J* = 12.5, 6.1, 3.3 Hz, 1H), 3.56 (d, *J* = 2.0 Hz, 1H), 3.47 (dd, *J* = 8.8, 3.6 Hz, 1H), 3.26–3.32 (m, 2H), 2.29 (s, 3H), 1.25 (d, *J* = 6.4 Hz, 3H); ^13^C-NMR (100 MHz, CDCl_3_) *δ* 195.0, 153.6, 149.0, 144.3, 133.4, 130.0, 127.8, 110.2, 89.3, 71.8, 56.0, 55.0, 45.8, 21.4, 20.9; HRMS (ESI+) calcd for [C_21_H_25_NO_6_S]^+^: *m*/*z* 420.1475, found 420.1464.

*1-(4-Fluorophenyl)-2-[(2S,4S)-4-Methyl-3-tosyloxazolidin-2-yl]ethanone* (**4i**). 51% yield; white solid; IR (CH_2_Cl_2_) *ν*_max_ 3069.4, 2981.0, 1686.4, 1597.3, 1349.2, 1164.7 cm^–1^; ^1^H-NMR (400 MHz, CDCl_3_) *δ* 8.00 (q, *J* = 1.1 Hz, 2H), 7.72 (d, *J* = 8.4 Hz, 2H), 7.31 (d, *J* = 8.0 Hz, 2H), 7.12 (t, *J* = 8.8 Hz, 2H), 5.46 (dd, *J* = 8.8, 2.0 Hz, 1H), 3.67–3.74 (m, 2H), 3.55 (dd, *J* = 8.8, 3.6 Hz, 1H), 3.35–3.41 (m, 2H), 2.39 (s, 3H), 1.34 (d, *J* = 6.4 Hz, 3H); ^13^C-NMR (100 MHz, CDCl_3_) *δ* 195.0, 167.2, 164.7, 144.4, 133.4, 133.1, 131.0, 130.1, 127.9, 115.9, 89.0, 71.9, 55.0, 46.2, 21.5, 21.1; HRMS (ESI+) calcd for [C_19_H_20_FNO_4_S]^+^: *m*/*z* 378.1170, found 378.1170.

*2-[(2S,4S)-4-Methyl-3-tosyloxazolidin-2-yl]-1-(naphth-1-yl)ethanone* (**4j**). 84% yield; brown oil; IR (CH_2_Cl_2_) *ν*_max_ 3051.4, 2980.3, 1678.6, 1596.0, 1348.9, 1164.0 cm^–1^; ^1^H-NMR (400 MHz, CDCl_3_) *δ* 8.73 (d, *J* = 8.4 Hz, 1H), 7.97 (d, *J* = 7.6 Hz, 2H), 7.84 (d, *J* = 8.0 Hz, 1H), 7.75 (d, *J* = 8.0 Hz, 2H), 7.47–7.59 (m, 3H), 7.28 (d, *J* = 8.0 Hz, 2H), 5.59 (dd, *J* = 8.6, 2.2 Hz, 1H), 3.89 (dd, *J* = 16.4, 2.4 Hz, 1H), 3.75 (ddd, *J* = 8.1, 4.5, 1.9 Hz, 1H), 3.56 (dd, *J* = 9.0, 3.4 Hz, 1H), 3.50 (dd, *J* = 16.8, 8.8 Hz, 1H), 3.39 (dd, *J* = 9.0, 6.2 Hz, 1H), 2.35 (s, 3H), 1.36 (d, *J* = 6.8 Hz, 3H); ^13^C-NMR (100 MHz, CDCl_3_) *δ* 200.2, 144.4, 134.9, 134.0, 133.5, 133.3, 130.2, 130.0, 128.6, 128.1, 126.6, 126.0, 124.5, 89.4, 71.9, 55.1, 49.4, 21.5, 21.1; HRMS (ESI+) calcd for [C_23_H_23_NO_4_S]^+^: *m*/*z* 410.1421, found 410.1421. 

*2-[(2S,4S)-4-Methyl-3-tosyloxazolidin-2-yl]-1-(thien-2-yl)ethanone* (**4k**). 49% yield; brown solid; IR (CH_2_Cl_2_) *ν*_max_ 3102.9, 2980.4, 1658.8, 1597.7, 1348.9, 1164.0 cm^–1^; ^1^H-NMR (400 MHz, CDCl_3_) *δ* 7.78 (dd, *J* = 3.8, 1.0 Hz, 1H), 7.72 (d, *J* = 8.0 Hz, 2H), 7.65 (dd, *J* = 5.0, 1.0 Hz, 1H), 7.32 (d, *J* = 8.0 Hz, 2H), 7.13 (dd, *J* = 4.8, 4.0 Hz, 1H), 5.45 (dd, *J* = 7.6, 3.2 Hz, 1H), 3.72 (ddd, *J* = 8.0, 4.4, 1.8 Hz, 1H), 3.63 (dd, *J* = 16.0, 2.0 Hz, 1H), 3.56 (dd, *J* = 8.8, 3.6 Hz, 1H), 3.32–3.41 (m, 2H), 2.40 (s, 3H), 1.34 (d, *J* = 6.4 Hz, 3H); ^13^C-NMR (100 MHz, CDCl_3_) *δ* 189.1, 144.4, 144.0, 134.4, 133.4, 132.9, 130.0, 128.4, 127.9, 89.0, 71.9, 55.0, 46.9, 21.6, 21.0; HRMS (ESI+) calcd for [C_17_H_19_NO_4_S_2_]^+^: *m*/*z* 366.0828, found 366.0822.

*Methyl 2-[(2S,4R)-4-benzyl-3-tosyloxazolidin-2-yl]acetate* (**5a**). 66% yield; white solid; IR (CH_2_Cl_2_) *ν*_max_ 3028.3, 2952.2, 1740.7, 1597.9, 1351.7, 1163.9 cm^–1^; ^1^H-NMR (400 MHz, CDCl_3_) *δ* 7.77 (d, *J* = 8.4 Hz, 2H), 7.18–7.34 (m, 7H), 5.33 (dd, *J* = 8.8, 2.8 Hz, 1H), 3.83 (ddd, *J* = 12.8, 6.4, 3.2 Hz, 1H), 3.72 (dd, *J* = 9.2, 3.6 Hz, 1H), 3.69 (s, 3H), 3.22 (dd, *J* = 9.2, 6.4 Hz, 1H), 3.13 (dd, *J* = 13.6, 3.6 Hz, 1H), 3.06 (dd, *J* = 15.8, 3.0 Hz, 1H), 2.84 (dd, *J* = 13.2, 10.0 Hz, 1H), 2.60 (dd, *J* = 15.8, 8.6 Hz, 1H), 2.40 (s, 3H); ^13^C-NMR (100 MHz, CDCl_3_) *δ* 169.9, 144.5, 137.0, 133.6, 130.1, 129.6, 128.7, 127.9, 126.9, 89.0, 69.1, 60.5, 51.9, 41.8, 40.9, 21.6; HRMS (ESI+) calcd for [C_20_H_23_NO_5_S]^+^: *m*/*z* 390.1370, found 390.1364.

*Ethyl 2-[(2R,4S)-4-benzyl-3-tosyloxazolidin-2-yl]acetate* (**5b**). 81% yield; white solid; IR (CH_2_Cl_2_) *ν*_max_ 3028.3, 2983.2, 1736.0, 1597.9, 1352.0, 1164.1 cm^–1^; ^1^H-NMR (400 MHz, CDCl_3_) *δ* 7.75 (dd, *J* = 8.4 Hz, 2H), 7.30 (d, *J* = 8.4 Hz, 2H), 7.26 (d, *J* = 7.6 Hz, 2H), 7.18 (d, *J* = 8.0 Hz, 3H), 5.33 (dd, *J* = 8.8, 2.8 Hz, 1H), 4.14 (q, *J* = 7.2 Hz, 2H), 3.82 (ddd, *J* = 13.0, 6.6, 3.2 Hz, 1H), 3.70 (dd, *J* = 9.2, 3.2 Hz, 1H), 3.20 (dd, *J* = 9.2, 6.0 Hz, 1H), 3.13 (dd, *J* = 13.4, 3.8 Hz, 1H), 3.05 (dd, *J* = 15.8, 3.0 Hz, 1H), 2.83 (dd, *J* = 13.4, 9.8 Hz, 1H), 2.37 (s, 3H), 1.24 (t, *J* = 7.2 Hz, 3H); ^13^C-NMR (100 MHz, CDCl_3_) *δ* 169.5, 144.5, 137.1, 133.6, 130.1, 129.6, 128.7, 127.9, 126.9, 89.1, 69.1, 60.7, 60.4, 42.0, 41.0, 21.5, 14.2; HRMS (ESI+) calcd for [C_21_H_25_NO_5_S]^+^: *m*/*z* 404.1526, found 404.1528.

*Benzyl 2-[(2S,4R)-4-benzyl-3-tosyloxazolidin-2-yl]acetate* (**5c**). 76% yield; yellow oil; IR (CH_2_Cl_2_) *ν*_max_ 3063.3, 3030.3, 2948.4, 1737.7, 1597.8, 1352.0, 1164.0 cm^–1^; ^1^H-NMR (400 MHz, CDCl_3_) *δ* 7.81 (d, *J* = 8.0 Hz, 2H), 7.23–7.42 (m, 12H), 5.44 (dd, *J* = 8.4, 3.2 Hz, 1H), 5.20 (s, 2H), 3.89 (ddd, *J* = 12.9, 6.5, 3.1 Hz, 1H), 3.78 (dd, *J* = 8.4, 5.2 Hz, 1H), 3.27 (dd, *J* = 9.2, 6.4 Hz, 1H), 3.16–3.21 (m, 2H), 2.88 (dd, *J* = 13.6, 10.0 Hz, 1H), 2.73 (dd, *J* = 16.0, 8.4 Hz, 1H), 2.43 (s, 3H); ^13^C-NMR (100 MHz, CDCl_3_) *δ* 169.4, 144.6, 137.1, 135.8, 133.7, 130.2, 130.1, 129.6, 128.8, 128.7, 128.5, 128.3, 128.0, 127.0, 89.2, 69.2, 66.7, 60.6, 42.1, 41.0, 21.6; HRMS (ESI+) calcd for [C_26_H_27_NO_5_S]^+^: *m*/*z* 466.1683, found 466.1687.

*Phenyl 2-[(2S,4R)-4-benzyl-3-tosyloxazolidin-2-yl]acetate* (**5d**). 5% yield; yellow oil; IR (CH_2_Cl_2_) *ν*_max_ 3028.5, 2924.3, 1712.3, 1589.8, 1325.3, 1362.2 cm^–1^; ^1^H-NMR (400 MHz, CDCl_3_) *δ* 7.80 (d, *J* = 8.4 Hz, 2H), 7.05–7.42 (m, 12H), 5.42 (dd, *J* = 8.0, 3.2 Hz, 1H), 3.86 (ddd, *J* = 13.1, 6.3, 3.3 Hz, 1H), 3.79 (dd, *J* = 9.2, 3.2 Hz, 1H), 3.33 (dd, *J* = 16.0, 3.2 Hz, 1H), 3.27 (dd, *J* = 9.0, 6.2 Hz, 1H), 3.20 (dd, *J* = 13.6, 3.6 Hz, 1H), 2.82–2.92 (m, 3H), 2.43 (s, 3H); ^13^C-NMR (100 MHz, CDCl_3_) *δ* 167.0, 159.8, 155.7, 143.4, 137.5, 136.3, 129.7, 127.0, 128.0, 101.1, 64.5, 54.1, 38.8, 21.5; HRMS (ESI+) calcd for [C_25_H_25_NO_5_S]^+^: *m*/*z* 452.1526, found 452.1515. 

*1-[(2S,4S)-4-Benzyl-3-tosyloxazolidin-2-yl]propan-2-one* (**5e**). 45% yield; yellow oil; IR (CH_2_Cl_2_) *ν*_max_ 3062.3, 2924.8, 1716.7, 1598.0, 1349.8, 1163.6 cm^–1^; ^1^H-NMR (400 MHz, CDCl_3_) *δ* 7.74 (d, *J* = 8.4 Hz, 2H), 7.32 (d, *J* = 8.0 Hz, 2H), 7.29 (d, *J* = 7.6 Hz, 2H), 7.19–7.22 (m, 3H), 5.27 (dd, *J* = 8.4, 2.4 Hz, 1H), 3.80 (ddd, *J* = 9.4, 6.2, 3.2 Hz, 1H), 3.69 (dd, *J* = 9.2, 2.8 Hz, 1H), 3.10–3.17 (m, 3H), 2.83 (dd, *J* = 13.6, 10.0 Hz, 1H), 2.72 (dd, *J* = 16.8, 8.4 Hz, 1H), 2.40 (s, 3H), 2.13 (s, 3H); ^13^C- NMR (100 MHz, CDCl_3_) *δ* 205.1, 144.5, 137.1, 133.5, 13071, 129.7, 128.7, 127.9, 126.9, 88.6, 69.0, 60.3, 50.2, 41.0, 30.8, 21.6; HRMS (ESI+) calcd for [C_20_H_23_NO_4_S]^+^: *m*/*z* 374.1421, found 374.1413.

*2-[(2S,4R)-4-Benzyl-3-tosyloxazolidin-2-yl]-1-phenylethanone* (**5f**). 71% yield; white solid; IR (CH_2_Cl_2_) *ν*_max_ 3060.0, 3027.9, 2924.2, 1687.4, 1597.2, 1349.6, 1163.3 cm^–1^; ^1^H-NMR (400 MHz, CDCl_3_) *δ* 7.92 (d, *J* = 7.2 Hz, 2H), 7.80 (d, *J* = 8.4 Hz, 2H), 7.24–7.58 (m, 9H), 5.55 (dd, *J* = 8.8, 2.0 Hz, 1H), 3.89 (ddd, *J* = 9.4, 6.2, 3.4 Hz, 1H), 3.75 (dd, *J* = 9.6, 3.2 Hz, 1H), 3.63 (dd, *J* = 16.8, 2.0 Hz, 1H), 3.13–3.28 (m, 3H), 2.97 (dd, *J* = 13.4, 9.4 Hz, 1H), 2.40 (s, 3H); ^13^C-NMR (100 MHz, CDCl_3_) *δ* 196.6, 144.5, 137.1 ,136.6, 133.5, 130.1, 130.0, 129.1, 128.7, 128.3, 128.0, 127.0, 89.2, 69.0, 60.2, 45.6, 40.8, 21.6; HRMS (ESI+) calcd for [C_25_H_25_NO_4_S]^+^: *m*/*z* 436.1577, found 436.1576.

*2-[(2R,4R)-4-Benzyl-3-tosyloxazolidin-2-yl]-1-(3,4-dichlorophenyl)ethanone* (**5g**). 30% yield; white solid; IR (CH_2_Cl_2_) *ν*_max_ 3063.5, 3028.4, 2924.0, 1691.4, 1597.7, 1350.47, 1164.0 cm^–1^; ^1^H-NMR (400 MHz, CDCl_3_) *δ* 7.94 (d, *J* = 2.0 Hz, 1H), 7.78 (d, *J* = 8.4 Hz, 2H), 7.72 (dd, *J* = 8.4, 2.0 Hz, 1H), 7.54 (d, *J* = 8.4 Hz, 2H), 7.25–7.37 (m, 8H), 5.46 (dd, *J* = 8.6, 2.2 Hz, 1H), 3.87 (ddd, *J* = 9.1, 6.1, 3.1 Hz, 1H), 3.76 (dd, *J* = 9.2, 3.2 Hz, 1H), 3.51 (dd, *J* = 16.8, 2.0 Hz, 1H), 3.28 (dd, *J* = 9.2, 6.4 Hz, 1H), 2.96–3.12 (m, 3H), 2.43 (s, 3H); ^13^C-NMR (100 MHz, CDCl_3_) *δ* 194.4, 144.6, 138.0, 136.9, 136.0, 133.4, 133.3, 130.8, 130.3, 130.1, 128.7, 127.9, 127.3, 127.0, 88.9, 69.0, 60.0, 45.5, 40.6, 21.6; HRMS (ESI+) calcd for [C_25_H_23_Cl_2_NO_4_S]^+^: *m*/*z* 504.0798, found 504.0802. 

*2-[(2R,4R)-4-Benzyl-3-tosyloxazolidin-2-yl]-1-(3,4-dimethoxyphenyl)ethanone* (**5h**). 98% yield; yellow oil; IR (CH_2_Cl_2_) *ν*_max_ 3062.0, 3027.3, 2936.2, 1672.8, 1595.7, 1347.3, 1163.2 cm^–1^; ^1^H-NMR (400 MHz, CDCl_3_) *δ* 7.73 (d, *J* = 8.0 Hz, 2H), 7.45–7.54 (m, 2H), 7.24 (t, *J* = 7.2 Hz, 2H), 7.17 (d, *J* = 14.0 Hz, 2H), 6.90–6.93 (m, 1H), 6.84 (d, *J* = 8.4 Hz, 1H), 5.47 (dd, *J* = 8.8, 2.0 Hz, 1H), 3.85 (s, 3H), 3.83 (s, 3H), 3.68 (dd, *J* = 9.0, 3.4 Hz, 1H), 3.52 (dd, *J* = 16.4, 1.6 Hz, 1H), 3.19 (dd, *J* = 9.2, 6.4 Hz, 1H), 3.04–3.13 (m, 1H), 2.88 (dd, *J* = 13.4, 9.4 Hz, 1H), 2.31 (s, 3H); ^13^C-NMR (100 MHz, CDCl_3_) *δ* 195.1, 153.6, 149.0, 144.4, 143.0, 137.5, 137.3, 137.0, 133.4, 130.1, 130.0, 129.9, 129.8, 126.8, 110.1, 89.4, 68.9, 56.8, 56.1, 55.9, 45.1, 40.6, 21.5, 21.0; HRMS (ESI+) calcd for [C_27_H_29_NO_6_S]^+^: *m*/*z* 496.1788, found 496.1779.

*2-[(2R,4R)-4-Benzyl-3-tosyloxazolidin-2-yl]-1-(4-fluorophenyl)ethanone* (**5i**). 51% yield; yellow solid; IR (CH_2_Cl_2_) *ν*_max_ 3063.5, 3028.6, 2924.9, 1686.4, 1597.2, 1350.0, 1163.9 cm^–1^; ^1^H-NMR (400 MHz, CDCl_3_) *δ* 7.93 (dd, *J* = 8.8, 5.6 Hz, 2H), 7.79 (d, *J* = 8.0 Hz, 2H), 7.10–7.34 (m, 9H), 5.50 (dd, *J* = 8.8, 2.0 Hz, 1H), 3.87 (ddd, *J* = 9.3, 6.3, 3.1 Hz, 1H), 3.75 (dd, *J* = 9.2, 3.2 Hz, 1H), 3.52 (dd, *J* = 16.8, 2.0 Hz, 1H), 3.25 (dd, *J* = 9.2, 6.0 Hz, 1H), 2.98–3.18 (m, 2H), 2.95 (dd, *J* = 13.6, 9.6 Hz, 1H), 2.40 (s, 3H); ^13^C-NMR (100 MHz, CDCl_3_) *δ* 195.0, 167.2, 164.7, 144.5, 137.0, 133.5, 133.1, 131.1, 131.0, 130.2, 130.0, 128.7, 128.0, 127.0, 115.8, 89.2, 69.0, 60.2, 45.5, 40.8, 21.6; HRMS (ESI+) calcd for [C_25_H_24_FNO_4_S]^+^: *m*/*z* 454.1483, found 454.1489.

*2-[(2R,4R)-4-Benzyl-3-tosyloxazolidin-2-yl]-1-(naphth-1-yl)ethanone* (**5j**). 96% yield; yellow oil; IR (CH_2_Cl_2_) *ν*_max_ 3060.6, 3028.6, 2924.4, 1678.7, 1596.3, 1349.6, 1163.4 cm^–1^; ^1^H-NMR (400 MHz, CDCl_3_) *δ* 8.82 (d, *J* = 8.4 Hz, 1H), 7.99 (d, *J* = 8.0 Hz, 1H), 7.84–7.90 (m, 4H), 7.50–7.62 (m, 3H), 7.22–7.35 (m, 7H), 5.66 (dd, *J* = 8.6, 2.2 Hz, 1H), 3.94 (ddd, *J* = 9.3, 6.3, 3.1 Hz, 1H), 3.78–3.85 (m, 2H), 3.34 (dd, *J* = 16.8, 8.8 Hz, 1H), 3.28 (dd, *J* = 9.2, 6.0 Hz, 1H), 3.18 (dd, *J* = 13.4, 3.4 Hz, 1H), 2.98 (dd, *J* = 13.6, 9.2 Hz, 1H), 2.36 (s, 3H); ^13^C-NMR (100 MHz, CDCl_3_) *δ* 200.1, 144.5, 137.1, 134.9, 134.0, 133.6, 133.4, 130.3, 130.2, 130.0, 128.9, 128.7, 128.6, 128.2, 128.0, 127.0, 126.6, 126.0, 127.5, 89.7, 69.1, 60.4, 48.8, 40.9, 21.6; HRMS (ESI+) calcd for [C_29_H_27_NO_4_S]^+^: *m*/*z* 486.1734, found 486.1729.

*2-[(2R,4R)-4-Benzyl-3-tosyloxazolidin-2-yl]-1-(thien-2-yl)ethanone* (**5k**). 41% yield; brown solid; IR (CH_2_Cl_2_) *ν*_max_ 3105.0, 3028.7, 2918.5, 1660.9, 1597.4, 1350.3, 1163.1 cm^–1^; ^1^H-NMR (400 MHz, CDCl_3_) *δ* 7.79 (d, *J* = 8.0 Hz, 2H), 7.69 (dd, *J* = 3.8, 1.0 Hz, 1H), 7.66 (dd, *J* = 5.0, 1.0 Hz, 1H), 7.33 (t, *J* = 7.2 Hz, 4H), 7.25 (d, *J* = 6.8 Hz, 3H), 7.14 (t, *J* = 2.6 Hz, 1H), 5.47 (dd, *J* = 8.6, 2.2 Hz, 1H), 3.86 (ddd, *J* = 9.4, 6.2, 3.2 Hz, 1H), 3.76 (dd, *J* = 9.2, 3.2 Hz, 1H), 3.54 (dd, *J* = 16.2, 2.2 Hz, 1H), 3.25 (dd, *J* = 9.2, 6.4 Hz, 1H), 3.09–3.15 (m, 2H), 2.94 (dd, *J* = 13.6, 9.6 Hz, 1H), 2.42 (s, 3H); ^13^C- NMR (100 MHz, CDCl_3_) *δ* 189.2, 144.6, 144.1, 137.1, 134.5, 134.4 ,133.5, 132.9, 130.2, 130.0, 128.7, 128.3, 128.0, 127.0, 89.2, 69.0, 60.2, 46.2, 40.7, 21.6; HRMS (ESI+) calcd for [C_23_H_23_NO_4_S_2_]^+^: *m*/*z* 442.1141, found 442.1119.

*Methyl 2-[(2S,4R)-4-(hydroxymethyl)-3-tosyloxazolidin-2-yl]acetate* (**6a**). 38% yield; yellow oil; IR (CH_2_Cl_2_) *ν*_max_ 3532.4, 2953.5, 1738.3. 1597.5. 1349.3. 1164.0 cm^–1^; ^1^H-NMR (400 MHz, CDCl_3_) *δ* 7.73 (d, *J* = 8.4 Hz, 2H), 7.34 (d, *J* = 8.4 Hz, 2H), 5.31 (dd, *J* = 8.2, 3.4 Hz, 1H), 3.87 (dd, *J* = 9.0, 3.4 Hz, 1H), 3.63–3.69 (m, 6H), 3.44 (dd, *J* = 9.0, 6.2 Hz, 1H), 3.03 (dd, *J* = 15.6, 3.6 Hz, 1H), 2.75 (dd, *J* = 16.0, 8.0 Hz, 1H), 2.41 (s, 3H); ^13^C-NMR (100 MHz, CDCl_3_) *δ* 170.0, 144.8, 133.0, 130.2, 128.0, 127.0, 89.1, 67.6, 63.1, 60.2, 52.0, 41.3, 21.6; HRMS (ESI+) calcd for [C_14_H_19_NO_6_S]^+^: *m*/*z* 330.1006, found 330.0995.

*Ethyl 2-[(2S,4R)-4-(hydroxymethyl)-3-tosyloxazolidin-2-yl]acetate* (**6b**). 50% yield; brown oil; IR (CH_2_Cl_2_) *ν*_max_ 3531.9, 2982.6, 1732.1, 1597.6, 1350.5, 1164.6 cm^–1^; ^1^H-NMR (400 MHz, CDCl_3_) *δ* 7.73 (d, *J* = 8.4 Hz, 2H), 7.33 (dd, *J* = 8.0 Hz, 2H), 5.31 (dd, *J* = 8.2, 3.4 Hz, 1H), 4.14 (q, *J* = 7.2 Hz, 2H), 3.87 (dd, *J* = 9.4, 3.4 Hz, 1H), 3.63–3.70 (m, 3H), 3.44 (dd, *J* = 9.0, 6.2 Hz, 1H), 3.01 (dd, *J* = 15.8, 3.4 Hz, 2H), 2.73 (dd, *J* = 15.8, 8.2 Hz, 1H), 2.37–2.41 (m, 4H), 1.24 (t, *J* = 7.2 Hz, 3H); ^13^C- NMR (100 MHz, CDCl_3_) *δ* 169.6, 144.8, 133.1, 130.1, 128.0, 89.1, 67.6, 63.2, 61.0, 60.2, 41.5, 21.6, 14.1; HRMS (ESI+) calcd for [C_15_H_21_NO_6_S]^+^: *m*/*z* 344.1162, found 344.1186.

*Benzyl 2-[(2S,4R)-4-(hydroxymethyl)-3-tosyloxazolidin-2-yl]acetate* (**6c**). 37% yield; brown oil; IR (CH_2_Cl_2_) *ν*_max_ 3532.5, 3064.1, 2949.6, 1736.5, 1597.4, 1350.1, 1164.3 cm^–1^; ^1^H-NMR (400 MHz, CDCl_3_) *δ* 7.75 (d, *J* = 8.0 Hz, 2H), 7.33–7.37 (m, 7H), 5.38 (dd, *J* = 8.0, 3.6 Hz, 1H), 5.16 (s, 2H), 3.89 (dd, *J* = 9.2, 3.6 Hz, 1H), 3.64–3.73 (m, 3H), 3.46 (dd, *J* = 9.2, 6.4 Hz, 1H), 3.10 (dd, *J* = 15.6, 3.6 Hz, 1H), 2.83 (dd, *J* = 15.6, 8.0 Hz, 1H), 2.42 (s, 3H); ^13^C-NMR (100 MHz, CDCl_3_) *δ* 169.5, 144.8, 141.0, 135.5, 133.1, 130.2, 128.6, 128.3, 128.0, 127.0, 89.1, 67.6, 66.8, 65.1, 63.2, 60.2, 41.5, 21.6; HRMS (ESI+) calcd for [C_20_H_23_NO_6_S]^+^: *m*/*z* 406.1319, found 406.1327.

*Phenyl 2-[(2S,4R)-4-(hydroxymethyl)-3-tosyloxazolidin-2-yl]acetate* (**6d**). 8% yield; yellow oil; IR (CH_2_Cl_2_) *ν*_max_ 3515.0, 3064.8, 2526.4, 1755.0, 1594.6, 1324.6, 1163.1 cm^–1^; ^1^H-NMR (400 MHz, CDCl_3_) *δ* 7.77 (d, *J* = 3.2 Hz, 2H), 7.74 (d, *J* = 4.4 Hz, 2H), 7.05–7.15 (m, 5H), 5.48 (dd, *J* = 7.6, 4.0 Hz, 1H), 3.95 (dd, *J* = 9.0, 3.8 Hz, 1H), 3.72–3.76 (m, 2H), 3.46–3.57 (m, 3H), 3.31 (dd, *J* = 15.4, 3.8 Hz, 1H), 3.03 (dd, *J* = 15.6, 7.6 Hz, 1H), 2.45 (s, 3H); ^13^C-NMR (100 MHz, CDCl_3_) *δ* 168.1, 150.4, 144.9, 143.7, 133.0, 130.2, 130.1, 129.8, 129.4, 128.0, 127.1, 121.6, 118.0, 100.8, 89.1, 67.7, 63.3, 62.4, 54.1, 41.6, 21.6; HRMS (ESI+) calcd for [C_19_H_21_NO_6_S]^+^: *m*/*z* 392.1162, found 392.1187.

*1-[(2S,4R)-4-(Hydroxymethyl)-3-tosyloxazolidin-2-yl]propan-2-one* (**6e**). 29% yield; yellow oil; IR (CH_2_Cl_2_) *ν*_max_ 3516.2, 3060.0, 2953.8, 1715.3, 1597.3, 1348.3, 1163.9 cm^–1^; ^1^H NMR (400 MHz, CDCl_3_) *δ* 7.72 (d, *J* = 8.0 Hz, 2H), 7.34 (d, *J* = 8.0 Hz, 2H), 5.28 (dd, *J* = 8.0, 2.8 Hz, 1H), 3.84 (dd, *J* = 10.4, 2.8 Hz, 1H), 3.67 (s, 3H), 3.35–3.39 (m, 1H), 3.15 (dd, *J* = 16.8, 2.8 Hz, 1H), 2.92 (dd, *J* = 16.8, 8.0 Hz, 2H), 2.42 (s, 3H), 2.19 (s, 3H); ^13^C NMR (100 MHz, CDCl_3_) *δ* 205.3, 144.8, 132.9, 130.2, 128.0, 88.7, 67.7, 63.5, 60.0, 49.8, 30.9, 21.6; HRMS (ESI+) calcd for [C_14_H_19_NO_5_S]^+^: *m*/*z* 314.1057, found 314.1050.

*2-[(2S,4R)-4-(Hydroxymethyl)-3-tosyloxazolidin-2-yl]-1-phenylethanone* (**6f**). 23% yield; brown solid; IR (CH_2_Cl_2_) *ν*_max_ 3515.4, 3061.5, 2933.4, 1687.2, 1597.1, 1347.7, 1163.3 cm^–1^; ^1^H-NMR (400 MHz, CDCl_3_) *δ* 7.96 (d, *J* = 8.4 Hz, 2H), 7.75 (d, *J* = 8.4 Hz, 2H), 7.54–7.58 (m, 1H), 7.45 (t, *J* = 7.6 Hz, 2H), 7.34 (d, *J* = 8.0 Hz, 2H), 5.53 (dd, *J* = 8.4, 2.4 Hz, 1H), 3.88 (dd, *J* = 8.6, 2.2 Hz, 1H), 3.68–3.72 (m, 4H), 3.47 (dd, *J* = 16.8, 8.4 Hz, 1H), 3.39–3.42 (m, 1H), 2.41 (s, 3H); ^13^C-NMR (100 MHz, CDCl_3_) *δ* 196.7, 144.8, 136.5, 133.6, 133.0, 130.2, 130.0, 128.7, 128.3, 128.0, 89.3, 67.8, 63.5, 60.1, 45.5, 21.6; HRMS (ESI+) calcd for [C_19_H_21_NO_5_S]^+^: *m*/*z* 376.1213, found 376.1187.

*1-(3,4-Dichlorophenyl)-2-[(2S,4R)-4-(hydroxymethyl)-3-tosyloxazolidin-2-yl]ethanone* (**6g**). 12% yield; brown oil; IR (CH_2_Cl_2_) *ν*_max_ 3513.6, 3064.1, 2925.2, 1691.4, 1584.1, 1333.8, 1162.5 cm^–1^; ^1^H- NMR (400 MHz, CDCl_3_) *δ* 8.05 (d, *J* = 2.0 Hz, 1H), 7.80 (d, *J* = 7.6 Hz, 2H), 7.37 (d, *J* = 8.0 Hz, 2H), 7.28 (d, *J* = 7.6 Hz, 2H), 5.48 (dd, *J* = 8.2, 2.6 Hz, 1H), 3.88 (dd, *J* = 8.6, 2.2 Hz, 1H), 3.66–3.77 (m, 7H), 3.39–3.45 (m, 2H), 2.45 (s, 3H), 2.40–2.42 (m, 4H); ^13^C-NMR (100 MHz, CDCl_3_) *δ* 194.4, 145.0, 144.2, 138.2, 136.1, 133.5, 132.8, 131.0, 130.9, 128.0, 127.3, 127.2, 126.9, 126.4, 89.1, 60.1, 53.4, 21.6; HRMS (ESI+) calcd for [C_19_H_19_Cl_2_NO_5_S]^+^: *m*/*z* 444.0434, found 444.0388.

*1-(3,4-Dimethoxyphenyl)-2-[(2S,4R)-4-(hydroxymethyl)-3-tosyloxazolidin-2-yl]ethanone* (**6h**). 38% yield; yellow oil; IR (CH_2_Cl_2_) *ν*_max_ 3268.9, 3060.0, 2966.1, 1673.8, 1595.8, 1334.0, 1149.5 cm^–1^; ^1^H- NMR (400 MHz, CDCl_3_) *δ* 7.73 (d, *J* = 8.4 Hz, 2H), 7.49 (dd, *J* = 8.4, 2.0 Hz, 1H), 7.41 (d, *J* = 2.0 Hz, 1H), 7.22 (d, *J* = 8.0 Hz, 2H), 6.81 (d, *J* = 8.4 Hz, 1H), 6.07 (d, *J* = 9.6 Hz, 1H), 5.13 (t, *J* = 5.0 Hz, 1H), 3.88 (s, 3H), 3.73–3.84 (m, 6H), 3.27 (d, *J* = 9.6 Hz, 1H), 3.22 (d, *J* = 4.8 Hz, 2H), 2.34 (s, 3H); ^13^C-NMR (100 MHz, CDCl_3_) *δ* 194.6, 194.5, 153.6, 149.0, 148.9, 143.7, 143.5, 138.1, 137.7, 130.0, 129.8, 127.0, 126.8, 123.4, 110.1, 100.0, 70.5, 56.0, 47.8, 45.3, 43.1, 42.8, 21.5; HRMS (ESI+) calcd for [C_21_H_25_NO_7_S]^+^: *m*/*z* 436.1425, found 436.1418.

*1-(4-Fluorophenyl)-2-[(2S,4R)-4-(hydroxymethyl)-3-tosyloxazolidin-2-yl]ethanone* (**6i**). 23% yield; brown oil; IR (CH_2_Cl_2_) *ν*_max_ 3515.7, 3064.1, 2925.2, 1687.0, 1597.5, 1346.7, 1163.5 cm^–1^; ^1^H-NMR (400 MHz, CDCl_3_) *δ* 8.00 (dd, *J* = 8.8, 5.6 Hz, 2H), 7.75 (d, *J* = 8.4 Hz, 2H), 7.35 (d, *J* = 8.0 Hz, 2H), 7.12 (t, *J* = 8.6 Hz, 2H), 5.51 (dd, *J* = 8.4, 2.4 Hz, 1H), 3.88 (dd, *J* = 8.4, 2.0 Hz, 1H), 3.65–3.74 (m, 5H), 3.39–3.48 (m, 2H), 2.43 (s, 3H), 2.38–2.41 (m, 1H); ^13^C-NMR (100 MHz, CDCl_3_) *δ* 195.1, 144.9, 132.9, 132.4, 132.3, 131.1, 131.0, 130.2, 129.1, 128.0, 89.3, 67.8, 63.4, 60.1, 45.4, 22.6, 21.6; HRMS (ESI+) calcd for [C_19_H_20_FNO_5_S]^+^: *m*/*z* 394.1119, found 394.1100.

*2-[(2S,4R)-4-(Hydroxymethyl)-3-tosyloxazolidin-2-yl]-1-(naphth-1-yl)ethanone* (**6j**). 24% yield; brown oil; IR (CH_2_Cl_2_) *ν*_max_ 3515.8, 3052.0, 2957.9, 1678.1, 1509.0, 1346.9, 1163.1 cm^–1^; ^1^H-NMR (400 MHz, CDCl_3_) *δ* 8.70 (d, *J* = 8.0 Hz, 1H), 7.98 (dd, *J* = 9.0, 4.6 Hz, 2H), 7.77 (d, *J* = 8.4 Hz, 2H), 7.48–7.60 (m, 4H), 7.33 (d, *J* = 8.0 Hz, 2H), 5.58 (dd, *J* = 8.4, 2.8 Hz, 1H), 3.86–3.91 (m, 2H), 3.65–3.78 (m, 4H), 3.52 (dd, *J* = 16.4, 8.4 Hz, 1H), 3.42 (dd, *J* = 9.0, 6.2 Hz, 1H), 2.41 (s, 3H); ^13^C-NMR (100 MHz, CDCl_3_) *δ* 200.2, 144.8, 134.9, 134.0, 133.8, 133.4, 133.0, 130.2, 128.7, 128.6, 128.5, 125.9, 89.8, 76.8, 60.4, 48.7, 21.5, 21.1; HRMS (ESI+) calcd for [C_23_H_23_NO_5_S]^+^: *m*/*z* 426.1370, found 426.1365.

*2-[(2R,4R)-4-(Hydroxymethyl)-3-tosyloxazolidin-2-yl]-1-(thien-2-yl)ethanone* (**6k**). 58% yield; brown oil; IR (CH_2_Cl_2_) *ν*_max_ 3270.0, 3096.8, 2974.2, 1658.6, 1597.8, 1332.4, 1162.3 cm^–1^; ^1^H-NMR (400 MHz, CDCl_3_) *δ* 7.75 (dd, *J* = 8.2, 2.2 Hz, 2H), 7.67 (dd, *J* = 3.6, 1.2 Hz, 1H), 7.61 (d, *J* = 4.8 Hz, 1H), 7.25 (d, *J* = 9.6 Hz, 2H), 7.05–7.07 (m, 1H), 6.08 (d, *J* = 8.0 Hz, 1H), 5.11 (t, *J* = 4.4 Hz, 1H), 3.78 (q, *J* = 13.9 Hz, 3H), 3.20 (d, *J* = 5.2 Hz, 2H), 2.36 (s, 3H); ^13^C-NMR (100 MHz, CDCl_3_) *δ* 188.7, 144.2, 143.8, 143.6, 137.6, 134.6, 133.2, 133.1, 129.9, 128.4, 127.0, 99.4, 70.5, 69.9, 47.7, 44.3, 21.5; HRMS (ESI+) calcd for [C_17_H_19_NO_5_S_2_]^+^: *m*/*z* 382.0777, found 382.0781.

*Methyl 2-{(2S,3aS,7aS)-3-tosyloctahydrobenzo[d]oxazol-2-yl}acetate* (**7a**). 15% yield; white oil; IR (CH_2_Cl_2_) *ν*_max_ 2916.8, 2848.7, 1715.4, 1631.7, 1349.0, 1160.9 cm^–1^; ^1^H-NMR (400 MHz, CDCl_3_) *δ* 8.38 (d, *J* = 8.0 Hz, 2H), 8.13 (d, *J* = 8.0 Hz, 2H), 5.26 (dd, *J* = 8.0, 4.0 Hz, 1H), 3.68 (s, 3H), 3.42–3.48 (m, 1H), 3.15 (ddd, *J* = 13.0, 9.0, 3.0 Hz, 1H), 2.74 (dd, *J* = 16.0, 4.0 Hz, 1H), 2.46 (s, 3H), 2.29–2.34 (m, 1H), 1.96–1.98 (m, 1H), 1.77–1.81 (m, 2H), 1.40–1.43 (m, 1H), 1.30–1.35 (m, 4H); ^13^C-NMR (100 MHz, CDCl_3_) *δ* 171.4, 152.2, 147.5, 143.2, 136.5, 127.2, 121.1, 118.0, 110.0, 88.1, 60.2, 50.9, 39.3, 37.4, 29.7, 21.4; GCMS (EI+) calcd for [C_17_H_23_NO_5_S]: *m*/*z* 354.4, found 355.0.

*Ethyl 2-{(2S,3aS,7aS)-3-tosyloctahydrobenzo[d]oxazol-2-yl}acetate* (**7b**). 37% yield; white oil; IR (CH_2_Cl_2_) *ν*_max_ 2942.9, 1736.4, 1598.4, 1352.7, 1162.7 cm^–1^; ^1^H-NMR (400 MHz, CDCl_3_) *δ* 7.68 (d, *J* = 8.0 Hz, 2H), 7.34 (d, *J* = 8.0 Hz, 2H), 5.32 (dd, *J* = 9.6, 2.4 Hz, 1H), 4.15 (q, *J* = 7.2 Hz, 2H); ^13^C- NMR (100 MHz, CDCl_3_) *δ* 169.8, 144.4, 131.7, 130.0, 129.8, 128.2, 127.4, 88.3, 80.2, 64.5, 63.3, 60.7, 41.8, 29.6, 29.0, 24.4, 23.8, 23.3, 23.1, 21.6, 14.2; HRMS (ESI+) calcd for [C_18_H_25_NO_5_S]^+^: *m*/*z* 368.1526, found 368.1529.

*Benzyl 2-{(2S,3aS,7aS)-3-tosyloctahydrobenzo[d]oxazol-2-yl}acetate* (**7c**). 19% yield; yellow oil; IR (CH_2_Cl_2_) *ν*_max_ 3032.7, 2940.8, 2862.9, 1710.7, 1620.9, 1320.5, 1121.0 cm^–1^; ^1^H-NMR (400 MHz, CDCl_3_) *δ* 7.74 (d, *J* = 8.0 Hz, 2H), 7.36–7.38 (m, 5H), 7.23 (d, *J* = 8.0 Hz, 2H), 5.10 (s, 1H), 5.07 (s, 1H), 4.88 (s, 1H), 3.67 (ddd, *J* = 10.0, 6.0, 2.0 Hz, 1H), 3.42 (t, *J* = 8.0 Hz, 1H), 3.06–3.11 (m, 1H), 2.78 (t, *J* = 8.0 Hz, 1H), 2.39 (s, 3H), 2.25–2.27 (m, 1H), 1.98–2.02 (m, 1H), 1.74–1.83 (m, 1H), 1.66–1.70 (m, 1H), 1.53–1.61 (m, 1H), 1.24–1.31 (m, 3H); ^13^C-NMR (100 MHz, CDCl_3_) *δ* 169.9, 167.1, 167.0, 163.4, 160.6, 149.1, 145.1, 144.9, 143.1, 137.1, 136.4, 136.1, 135.5, 135.3, 135.2, 130.0, 129.9, 129.5, 128.4, 127.3, 102.9, 102.8, 98.0, 79.6, 77.3, 67.3, 67.1, 66.2, 65.6, 55.8, 51.5, 32.2, 31.2, 29.6, 28.8, 28.0, 25.0, 23.2, 21.7, 21.5; GCMS (EI+) calcd for [C_23_H_27_NO_5_S]: *m*/*z* 429.5, found 430.0.

*Phenyl 2-{(2S,3aS,7aS)-3-tosyloctahydrobenzo[d]oxazol-2-yl}acetate* (**7d**). 13% yield; white solid; IR (CH_2_Cl_2_) *ν*_max_ 3056.0, 2943.7, 1758.4, 1597.1, 1352.8, 1163.5 cm^–1^; ^1^H-NMR (400 MHz, CDCl_3_) *δ* 7.73 (d, *J* = 8.4 Hz, 2H), 7.36 (d, *J* = 8.4 Hz, 4H), 7.21–7.25 (m, 1H), 7.12 (dd, *J* = 5.0, 3.8 Hz, 2H), 5.44 (dd, *J* = 9.4, 2.6 Hz, 1H), 3.58–3.61 (m, 1H), 3.38 (dd, *J* = 16.2, 2.6 Hz, 1H), 3.05 (dd, *J* = 16.2, 9.0 Hz, 1H), 2.45–2.50 (m, 4H), 2.37 (ddd, *J* = 12.0, 8.0, 4.0 Hz, 1H), 1.84–2.07 (m, 1H), 1.81–1.84 (m, 2H), 1.51 (dd, *J* = 12.0, 3.6 Hz, 1H), 1.24–1.32 (m, 4H), 0.86–0.90 (m, 1H); ^13^C-NMR (100 MHz, CDCl_3_) *δ* 168.3, 150.5, 144.5, 131.6, 130.0, 129.4, 129.1, 128.2, 126.0, 121.6, 88.2, 80.6, 64.6, 42.1, 29.7, 29.1, 23.8, 23.3, 21.6; HRMS (ESI+) calcd for [C_22_H_25_NO_5_S]^+^: *m*/*z* 416.1526, found 416.1525.

*1-{(2S,3aS,7aS)-3-Tosyloctahydrobenzo[d]oxazol-2-yl}propan-2-one* (**7e**). 33% yield; yellow solid; IR (CH_2_Cl_2_) *ν*_max_ 3060.9, 2943.0, 1717.2, 1598.3, 1351.3, 1162.1 cm^–1^; ^1^H-NMR (400 MHz, CDCl_3_) *δ* 7.63 (d, *J* = 8.4 Hz, 2H), 7.32 (d, *J* = 8.0 Hz, 2H), 5.30 (dd, *J* = 9.2, 2.0 Hz, 1H), 3.42 (ddd, *J* = 10.9, 9.1, 2.7 Hz, 1H), 3.13 (dd, *J* = 13.6, 6.0 Hz, 1H), 2.95 (dd, *J* = 17.2, 9.2 Hz, 1H), 2.36–2.43 (m, 5H), 2.21–2.27 (m, 1H), 2.17 (s, 3H), 1.94–1.96 (m, 1H), 1.71–1.77 (m, 2H), 1.36–1.46 (m, 1H), 1.15–1.23 (m, 4H); ^13^C-NMR (100 MHz, CDCl_3_) *δ* 205.4, 144.4, 131.5, 130.0, 128.1, 127.3, 87.7, 80.3, 64.3, 50.3, 30.7, 29.6, 29.3, 29.0, 23.7, 23.3, 21.6; HRMS (ESI+) calcd for [C_17_H_23_NO_4_S]^+^: *m*/*z* 338.1421, found 338.1416.

*1-Phenyl-2-{(2S,3aS,7aS)-3-tosyloctahydrobenzo[d]oxazol-2-yl}ethanone* (**7f**). 32% yield; yellow oil; IR (CH_2_Cl_2_) *ν*_max_ 3056.0, 2942.4, 1687.0, 1597.5, 1350.5, 1162.7 cm^–1^; ^1^H-NMR (400 MHz, CDCl_3_) *δ* 7.99 (dd, *J* = 8.4, 1.2 Hz, 2H), 7.69 (d, *J* = 8.4 Hz, 2H), 7.54–7.58 (m, 1H), 7.47 (dd, *J* = 8.0, 7.2 Hz, 2H), 7.35 (d, *J* = 8.0 Hz, 2H), 5.57 (dd, *J* = 9.2, 2.0 Hz, 1H), 3.71 (dd, *J* = 17.2, 2.0 Hz, 1H), 3.48–3.54 (m, 2H), 2.49 (dd, *J* = 12.6, 2.2 Hz, 1H), 2.43 (s, 3H), 2.32 (ddd, *J* = 11.9, 8.7, 2.9 Hz, 1H), 1.81–2.02 (m, 1H), 1.75–1.81 (m, 2H), 1.50 (dd, *J* = 12.6, 3.0 Hz, 1H), 1.20–1.27 (m, 3H); ^13^C-NMR (100 MHz, CDCl_3_) *δ* 196.8, 144.5, 136.6, 133.4, 131.5, 130.0, 128.7, 128.3, 127.4, 88.3, 80.4, 64.4, 45.8, 29.6, 29.0, 23.8, 23.3, 21.6; HRMS (ESI+) calcd for [C_22_H_25_NO_4_S]^+^: *m*/*z* 400.1577, found 400.1582.

*1-(3,4-Dichlorophenyl)-2-(3-tosyloctahydrobenzo[d]oxazol-2-yl)ethanone* (**7g**). 18% yield; yellow solid; IR (CH_2_Cl_2_) *ν*_max_ 3068.0, 2946.6, 2863.7, 1685.3, 1583.9, 1351.1, 1163.7 cm^–1^; ^1^H-NMR (400 MHz, CDCl_3_) *δ* 8.06 (s, 1H), 7.81 (dd, *J* = 10.0, 2.0 Hz, 1H), 7.68 (d, *J* = 8.0 Hz, 2H), 7.55 (d, *J* = 8.0 Hz, 1H), 7.36 (d, *J* = 8.0 Hz, 2H), 5.50 (dd, *J* = 10.0, 2.0 Hz, 1H), 3.68 (dd, *J* = 16.0, 4.0 Hz, 1H), 3.51 (td, *J* = 10.0, 4.0 Hz, 1H), 3.45 (dd, *J* = 18.0, 10.0 Hz, 1H), 2.48 (dd, *J* = 14.0, 2.0 Hz, 1H), 2.44 (s, 3H), 2.31 (ddd, *J* = 12.0, 8.0, 4.0 Hz, 1H), 1.97–1.99 (m, 1H), 1.76–1.82 (m, 2H), 1.45–1.54 (m, 1H), 1.20–1.27 (m, 3H); ^13^C-NMR (100 MHz, CDCl_3_) *δ* 194.6, 144.6, 138.0, 136.1, 133.4, 130.9, 130.3, 129.9, 128.3, 128.2, 127.5, 127.4, 127.3, 88.0, 80.5, 77.3, 64.3, 45.9, 29.6, 29.0, 23.8, 23.3, 21.6; GCMS (EI+) calcd for [C_22_H_23_Cl_2_NO_4_S]: *m*/*z* 468.4, found 468.1.

*1-(3,4-Dimethoxyphenyl)-2-{(2S,3aS,7aS)-3-tosyloctahydrobenzo[d]oxazol-2-yl}ethanone* (**7h**) 93% yield; yellow solid; IR (CH_2_Cl_2_) *ν*_max_ 2938.9, 1672.7, 1596.0, 1348.4, 1161.1 cm^–1^; ^1^H-NMR (400 MHz, CDCl_3_) *δ* 7.61 (d, *J* = 8.4 Hz, 2H), 7.56 (dd, *J* = 8.4, 1.6 Hz, 1H), 7.46 (d, *J* = 2.0 Hz, 1H), 7.26 (d, *J* = 8.0 Hz, 2H), 7.16 (d, *J* = 8.0 Hz, 1H), 6.83 (d, *J* = 8.4 Hz, 1H), 5.48 (d, *J* = 7.6 Hz, 1H), 3.83 (s, 3H), 3.82 (s, 3H), 3.38–3.60 (m, 3H), 3.21 (ddd, *J* = 11.8, 7.8, 2.2 Hz, 1H), 2.21–2.40 (m, 5H), 1.84–1.92 (m, 2H), 1.61–1.70 (m, 3H), 1.37–1.46 (m, 2H), 1.11–1.18 (m, 4H); ^13^C-NMR (100 MHz, CDCl_3_) *δ* 195.4, 153.5, 149.0, 144.4, 143.2, 137.7, 131.4, 130.0, 129.7, 128.1, 127.0, 110.2, 88.5, 80.2, 72.9, 64.3, 59.6, 56.0, 54.3, 33.3, 31.5, 29.6, 29.0, 24.6, 23.7, 21.4; HRMS (ESI+) calcd for [C_22_H_23_Cl_2_NO_4_S]^+^: *m*/*z* 468.0798, found 468.0830.

*1-(4-Fluorophenyl)-2-{(2S,3aS,7aS)-3-tosyloctahydrobenzo[d]oxazol-2-yl}ethanone* (**7i**). 27% yield; yellow solid; IR (CH_2_Cl_2_) *ν*_max_ 2942.4, 1687.2, 1597.4, 1350.7, 1162.6 cm^–1^; ^1^H-NMR (400 MHz, CDCl_3_) *δ* 8.01 (t, *J* = 4.4 Hz, 2H), 7.69 (d, *J* = 8.0 Hz, 2H), 7.35 (d, *J* = 8.0 Hz, 2H), 7.14 (t, *J* = 8.8 Hz, 2H), 5.53 (dd, *J* = 9.2, 1.6 Hz, 1H), 3.69 (dd, *J* = 17.0, 1.8 Hz, 1H), 3.44–3.54 (m, 2H), 2.42–2.50 (m, 5H), 2.31 (ddd, *J* = 11.7, 8.9, 3.1 Hz, 1H), 1.97–2.00 (m, 1H), 1.75–1.81 (m, 2H), 1.50 (ddd, *J* = 18.1, 12.1, 6.1 Hz, 1H), 1.19–1.27 (m, 3H); ^13^C-NMR (100 MHz, CDCl_3_) *δ* 195.2, 167.1, 164.6, 144.5, 134.3, 133.1, 133.0, 132.0, 131.7, 131.0, 128.2, 115.9, 88.2, 80.4, 64.4, 45.7, 31.5, 29.7, 23.9, 23.3, 21.6; HRMS (ESI+) calcd for [C_22_H_24_FNO_4_S]^+^: *m*/*z* 418.1483, found 418.1496.

*1-(Naphth-1-yl)-2-{(2S,3aS,7aS)-3-tosyloctahydrobenzo[d]oxazol-2-yl}ethanone* (**7j**). 33% yield; brown oil; IR (CH_2_Cl_2_) *ν*_max_ 3056.0, 2942.5, 1678.0, 1596.5, 1350.8, 1162.5 cm^–1^; ^1^H-NMR (400 MHz, CDCl_3_) *δ* 8.75 (d, *J* = 8.4, 1H), 7.99 (d, *J* = 6.8 Hz, 2H), 7.86 (d, *J* = 8.0 Hz, 1H), 7.72 (d, *J* = 8.4 Hz, 2H), 7.51–7.61 (m, 4H), 7.33 (d, *J* = 8.0 Hz, 2H), 5.63 (dd, *J* = 9.2, 2.0 Hz, 1H), 3.88 (dd, *J* = 16.8, 2.0 Hz, 1H), 3.54–3.62 (m, 2H), 2.51 (d, *J* = 12.0 Hz, 1H), 2.41 (s, 3H), 2.35 (ddd, *J* = 11.7, 9.1, 3.1 Hz, 1H), 1.99–2.03 (m, 1H), 1.75–1.81 (m, 2H), 1.46–1.52 (m, 1H), 1.20–1.29 (m, 3H); ^13^C-NMR (100 MHz, CDCl_3_) *δ* 200.5, 144.5, 134.9, 134.0, 133.3, 131.5, 130.3, 130.0, 128.7, 128.5, 127.5, 127.4, 126.6, 124.4, 88.7, 80.4, 64.5, 48.9, 29.7, 29.1, 23.9, 23.8, 21.6; HRMS (ESI+) calcd for [C_26_H_27_NO_4_S]^+^: *m*/*z* 450.1734, found 450.1732.

*1-(Thien-2-yl)-2-{(2S,3aS,7aS)-3-tosyloctahydrobenzo[d]oxazol-2-yl}ethanone* (**7k**). 25% yield; brown solid; IR (CH_2_Cl_2_) *ν*_max_ 2943.0, 1659.0, 1598.0, 1350.8, 1162.2 cm^–1^; ^1^H-NMR (400 MHz, CDCl_3_) *δ* 7.80 (dd, *J* = 3.8, 1.0 Hz, 1H), 7.69 (d, *J* = 8.4 Hz, 2H), 7.65 (dd, *J* = 5.0, 1.0 Hz, 1H), 7.35 (d, *J* = 8.0 Hz, 2H), 7.14 (dd, *J* = 5.0, 3.8 Hz, 1H), 5.51 (dd, *J* = 9.2, 2.0 Hz, 1H), 3.63 (dd, *J* = 16.4, 2.0 Hz, 1H), 3.54 (ddd, *J* = 10.9, 8.9, 2.9 Hz, 1H), 3.44 (dd, *J* = 16.2, 8.6 Hz, 1H), 2.41–2.49 (m, 4H), 2.31 (ddd, *J* = 12.2, 8.6, 3.0 Hz, 1H), 1.97–1.99 (m, 1H), 1.75–1.81 (m, 2H), 1.44–1.50 (m, 1H), 1.16–1.27 (m, 3H); ^13^C-NMR (100 MHz, CDCl_3_) *δ* 189.4, 144.5, 144.0, 134.2, 132.6, 131.5, 130.0, 128.3, 88.3, 80.4, 64.4, 46.4, 29.6, 29.0, 23.8, 23.3, 21.6; HRMS (ESI+) calcd for [C_20_H_23_NO_4_S_2_]^+^: *m*/*z* 406.1141, found 406.1136.

*(2S,4S)-4-Isopropyl-3-tosyl-2-(tosylmethyl)oxazolidine* (**8a**). 30% yield; white solid; IR (CH_2_Cl_2_) *ν*_max_ 3064.1, 2963.9, 1597.1, 1350.3, 1318.9, 1163.7 cm^–1^; ^1^H-NMR (400 MHz, CDCl_3_) *δ* 7.82 (d, *J* = 8.4 Hz, 2H), 7.53 (d, *J* = 8.4 Hz, 2H), 7.37 (d, *J* = 8.0 Hz, 2H), 7.30 (d, *J* = 8.0 Hz, 2H), 5.13 (dd, *J* = 8.8, 1.2 Hz, 1H), 3.90 (dd, *J* = 14.2, 1.4 Hz, 1H), 3.74 (dd, *J* = 9.4, 2.2 Hz, 1H), 3.34–3.44 (m, 2H), 3.07 (dd, *J* = 9.2, 6.0 Hz, 1H), 2.46 (s, 3H), 2.42 (s, 3H), 1.78–1.87 (m, 1H), 0.94 (d, *J* = 6.8 Hz, 3H), 0.87 (d, *J* = 6.8 Hz, 3H); ^13^C-NMR (100 MHz, CDCl_3_) *δ* 145.0, 136.6, 132.9, 130.1 , 129.9, 128.3, 128.1, 128.0, 86.7, 68.4, 64.4, 61.6, 31.3, 21.7, 19,4, 18.2; HRMS (ESI+) calcd for [C_21_H_27_NO_5_S_2_]^+^: *m*/*z* 438.1403, found 438.1387.

*(2S,4S)-4-Methyl-3-tosyl-2-(tosylmethyl)oxazolidine* (**8b**). 68% yield; white oil; IR (CH_2_Cl_2_) *ν*_max_ 3063.2, 2983.5, 1597.6, 1351.3, 1164.6 cm^–1^; ^1^H-NMR (400 MHz, CDCl_3_) *δ* 7.80 (d, *J* = 8.4 Hz, 2H), 7.56 (d, *J* = 13.6 Hz, 2H), 7.35 (d, *J* = 8.0 Hz, 2H), 7.28 (d, *J* = 8.0 Hz, 2H), 5.17 (d, *J* = 9.2 Hz, 1H), 3.85 (d, *J* = 14.4 Hz, 1H), 3.63 (ddd, *J* = 11.1, 6.7, 2.7 Hz, 1H), 3.36–3.48 (m, 3H), 2.43 (s, 3H), 2.40 (s, 3H), 1.25 (d, *J* = 6.4 Hz, 3H); ^13^C-NMR (100 MHz, CDCl_3_) *δ* 145.0, 144.7, 136.7, 132.7, 130.1, 129.9, 128.3, 127.8, 86.8, 73, 61.6, 54.8, 21.7, 21.6, 20.7; HRMS (ESI+) calcd for [C_19_H_23_NO_5_S_2_]^+^: *m*/*z* 410.1090, found 410.1094.

*(2S,4S)-4-Benzyl-3-tosyl-2-(tosylmethyl)oxazolidine* (**8c**). 65% yield; yellow oil; IR (CH_2_Cl_2_) *ν*_max_ 3062.4, 3029.4, 2927.1, 1597.4, 1352.2, 1163.6 cm^–1^; ^1^H-NMR (400 MHz, CDCl_3_) *δ* 7.77 (d, *J* = 8.4 Hz, 2H), 7.59 (d, *J* = 8.0 Hz, 2H), 7.15–7.37 (m, 9H), 5.18 (dd, *J* = 9.2, 1.6 Hz, 1H), 3.73–3.81 (m, 2H), 3.66 (dd, *J* = 9.4, 3.4 Hz, 1H), 3.22 (dd, *J* = 9.2, 6.4 Hz, 1H), 3.08 (dd, *J* = 14.6, 9.4 Hz, 1H), 3.02 (dd, *J* = 13.6, 3.2 Hz, 1H), 2.84 (dd, *J* = 13.4, 9.4 Hz, 1H), 2.46 (s, 3H), 2.42 (s, 3H); ^13^C-NMR (100 MHz, CDCl_3_) *δ* 144.9, 136.7, 136.5, 132.8, 130.2, 129.8, 128.7, 128.3, 127.8, 127.0, 86.9, 69.5, 61.0, 59.8, 40.4, 21.7, 21.6; HRMS (ESI+) calcd for [C_25_H_27_NO_5_S_2_]^+^: *m*/*z* 486.1403, found 486.1403.

*[(2S,4R)-3-Tosyl-2-(tosylmethyl)oxazolidin-4-yl]methanol* (**8d**). 81% yield; white oil; IR (CH_2_Cl_2_) *ν*_max_ 3532.6, 3063.3, 2928.6, 1597.3, 1351.3, 1164.0 cm^–1^; ^1^H-NMR (400 MHz, CDCl_3_) *δ* 7.78 (d, *J* = 8.0 Hz, 2H), 7.58 (d, *J* = 8.4 Hz, 2H), 7.33 (d, *J* = 8.0 Hz, 2H), 7.30 (d, *J* = 8.0 Hz, 2H), 5.24 (d, *J* = 8.8 Hz, 1H), 3.76–3.83 (m, 2H), 3.53–3.65 (m, 4H), 3.38 (dd, *J* = 9.2, 6.4 Hz, 1H), 2.82 (s, 1H), 2.42 (s, 3H), 2.41 (s, 3H); ^13^C-NMR (100 MHz, CDCl_3_) *δ* 145.1, 145.0, 136.6, 132.4, 130.3, 13.2, 129.9, 129.8, 128.2, 127.9, 87.1, 68.0, 62.8, 60.9, 59.7, 21.7, 21.6; HRMS (ESI+) calcd for [C_19_H_23_NO_6_S_2_]^+^: *m*/*z* 426.1040, found 426.1042. 

*(2S,3aS,7aS)-3-Tosyl-2-(tosylmethyl)octahydrobenzo[d]oxazole* (**8e**). 37% yield; white oil; IR (CH_2_Cl_2_) *ν*_max_ 3056.0, 2942.9, 1597.4, 1352.8, 1161.4 cm^–1^; ^1^H-NMR (400 MHz, CDCl_3_) *δ* 7.82 (d, *J* = 8.4 Hz, 2H), 7.44 (d, *J* = 8.4 Hz, 2H), 7.36 (d, *J* = 8.0 Hz, 2H), 7.29 (d, *J* = 8.0 Hz, 2H), 5.13 (dd, *J* = 9.4, 1.4 Hz, 1H), 3.83 (dd, *J* = 14.8, 1.6 Hz, 1H), 3.54 (dd, *J* = 14.8, 9.6 Hz, 1H), 3.30 (ddd, *J* = 11.8, 8.2, 2.0 Hz, 1H), 2.45 (s, 3H), 2.42 (s, 3H), 2.19–2.27 (m, 1H), 1.71–1.85 (m, 4H), 1.33–1.43 (m, 1H), 1.15–1.24 (m, 3H); ^13^C-NMR (100 MHz, CDCl_3_) *δ* 144.8, 136.8, 130.9, 130.3, 130.0, 128.4, 86.0, 80.8, 64.1, 60.7, 29.7, 29.5, 23.6, 23.2, 21.6; HRMS (ESI+) calcd for [C_22_H_27_NO_5_S_2_]^+^: *m*/*z* 450.1403, found 450.1406.

## 4. Conclusions

We have generated a chemical library of 60 unique oxazolidines, which will be tested for biological activity. We have also expanded the scope of the possible pro-nucleophiles and electron-deficient acetylenes that can be employed in mixed double-Michael reactions, thereby enabling the syntheses of more-diverse oxazolidines.

## Figures and Tables

**Figure 1:**
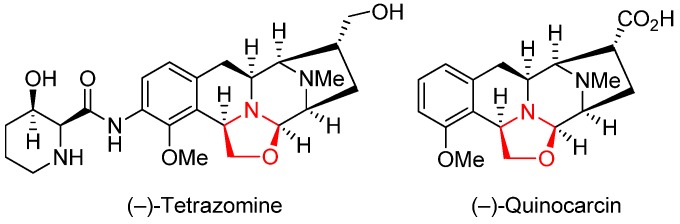
Oxazolidine-Containing Natural Products.

**Scheme 2:**
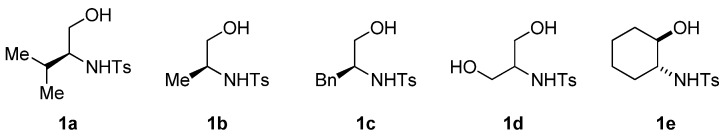
Amino-Alcohol Pro-Nucleophiles.

**Table 1:**
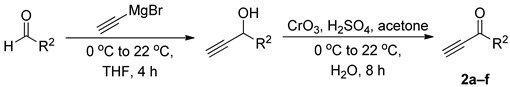
Synthesis of Electron-Deficient Acetylenes.

Entry	R^2^	Acetylene	Yield (%)
1	Ph	**2a**	83
2	*m*,*p*-Cl_2_C_6_H_3_	**2b**	76
3	*m*,*p*-(CH_3_O)_2_C_6_H_3_	**2c**	89
4	*p*-FC_6_H_4_	**2d**	76
5	1-naphthyl	**2e**	67
6	2-thienyl	**2f**	75

**Table 2:**
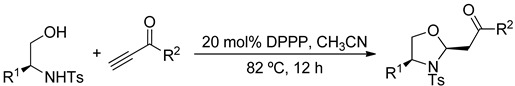
Synthesis of Trisubstituted Oxazolidines.

Entry	R^1^	R^2^	Oxazolidine	Yield (%)
1	*i*-Pr	OMe	(2*S*, 4*S*)-**3a**	59
2	*i*-Pr	OEt	(2*S*, 4*S*)-**3b**	75
3	*i*-Pr	OBn	(2*S*, 4*S*)-**3c**	63
4	*i*-Pr	OPh	(2*S*, 4*S*)-**3d**	22
5	*i*-Pr	Me	(2*S*, 4*S*)-**3e**	53
6	*i*-Pr	Ph	(2*S*, 4*S*)-**3f**	55
7	*i*-Pr	*m*,*p*-Cl_2_C_6_H_3_	(2*S*, 4*S*)-**3g**	28
8	*i*-Pr	*m*,*p*-(CH_3_O)_2_C_6_H_3_	(2*S*, 4*S*)-**3h**	58
9	*i*-Pr	*p*-FC_6_H_4_	(2*S*, 4*S*)-**3i**	63
10	*i*-Pr	1-naphthyl	(2*S*, 4*S*)-**3j**	70
11	*i*-Pr	2-thienyl	(2*S*, 4*S*)-**3k**	37
12	Me	OMe	(2*S*, 4*S*)-**4a**	68
13	Me	OEt	(2*S*, 4*S*)-**4b**	68
14	Me	OBn	(2*S*, 4*S*)-**4c**	64
15	Me	OPh	(2*S*, 4*S*)-**4d**	14
16	Me	Me	(2*S*, 4*S*)-**4e**	68
17	Me	Ph	(2*S*, 4*S*)-**4f**	57
18	Me	*m*,*p*-Cl_2_C_6_H_3_	(2*S*, 4*S*)-**4g**	41
19	Me	*m*,*p*-(CH_3_O)_2_C_6_H_3_	(2*S*, 4*S*)-**4h**	90
20	Me	*p*-FC_6_H_4_	(2*S*, 4*S*)-**4i**	27
21	Me	1-naphthyl	(2*S*, 4*S*)-**4j**	33
22	Me	2-thienyl	(2*S*, 4*S*)-**4k**	25
23	Bn	OMe	(2*S*, 4*S*)-**5a**	66
24	Bn	OEt	(2*S*, 4*S*)-**5b**	81
25	Bn	OBn	(2*S*, 4*S*)-**5c**	76
26	Bn	OPh	(2*S*, 4*S*)-**5d**	5
27	Bn	Me	(2*S*, 4*S*)-**5e**	45
28	Bn	Ph	(2*S*, 4*S*)-**5f**	71
29	Bn	*m*,*p*-Cl_2_C_6_H_3_	(2*S*, 4*S*)-**5g**	30
30	Bn	*m*,*p*-(CH_3_O)_2_C_6_H_3_	(2*S*, 4*S*)-**5h**	98
31	Bn	*p*-FC_6_H_4_	(2*S*, 4*S*)-**5i**	51
32	Bn	1-naphthyl	(2*S*, 4*S*)-**5j**	96
33	Bn	2-thienyl	(2*S*, 4*S*)-**5k**	41
34	CH_2_OH	OMe	(±)-**6a**	38
35	CH_2_OH	OEt	(±)-**6b**	50
36	CH_2_OH	OBn	(±)-**6c**	37
37	CH_2_OH	OPh	(±)-**6d**	8
38	CH_2_OH	Me	(±)-**6e**	29
39	CH_2_OH	Ph	(±)-**6f**	23
40	CH_2_OH	*m*,*p*-Cl_2_C_6_H_3_	(±)-**6g**	12
41	CH_2_OH	*m*,*p*-(CH_3_O)_2_C_6_H_3_	(±)-**6h**	38
42	CH_2_OH	*p*-FC_6_H_4_	(±)-**6i**	23
43	CH_2_OH	1-naphthyl	(±)-**6j**	24
44	CH_2_OH	2-thienyl	(±)-**6k**	58

**Table 3:**
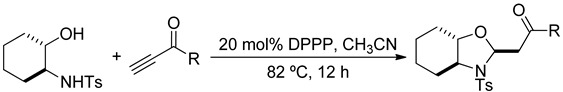
Synthesis of Octahydrobenzoxazolidines.

Entry	R	Oxazolidine	Yield (%)
1	OMe	(±)-**7a**	15
2	OEt	(±)-**7b**	37
3	OBn	(±)-**7c**	19
4	OPh	(±)-**7d**	13
5	Me	(±)-**7e**	33
6	Ph	(±)-**7f**	32
7	*m*,*p*-Cl_2_C_6_H_3_	(±)-**7g**	18
8	*m*,*p*-(CH_3_O)_2_C_6_H_3_	(±)-**7h**	93
9	*p*-FC_6_H_4_	(±)-**7i**	27
10	1-naphthyl	(±)-**7j**	33
11	2-thienyl	(±)-**7k**	25

**Table 4:**
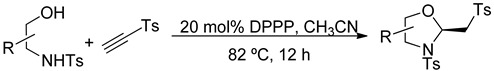
Tosyl Acetylene as Mixed Double-Michael Acceptor.

Entry	Pro-Nucleophile	Oxazolidine	Yield (%)
1	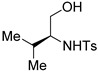	(2*S*, 4*S*)-**8a**	30
2	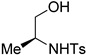	(2*S*, 4*S*)-**8b**	68
3	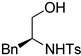	(2*S*, 4*S*)-**8c**	65
4	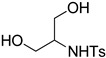	(**±**)**-8d**	81
5	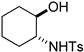	(**±**)**-8e**	37
